# Optimal dose of resistance training to improve handgrip strength in older adults with sarcopenia: a systematic review and Bayesian model-based network meta-analysis

**DOI:** 10.3389/fphys.2025.1564988

**Published:** 2025-07-02

**Authors:** Li Hua-Rui, Huang Shouliang, Yv Zhengze, Jiang Ning, Li Peihua, Zhai Yifei, Peng Fenglin

**Affiliations:** ^1^ College of Sport and Health, Guangxi Normal University, Guilin, China; ^2^ Sports Science Institution, Nanjing University, Nanjing, Jiangsu, China

**Keywords:** older adults, sarcopenia, resistance training, optimal dose, review

## Abstract

**Background:**

Sarcopenia is prevalent in older adults and affects their quality of life and overall health, low handgrip strength is one of the main manifestations of sarcopenia. Resistance training is an effective intervention for improving muscle strength in older adults, but the optimal dose of resistance training remains unclear. Therefore, the aim of this meta-analysis was to investigate the dose-response relationship between different doses of resistance training and grip strength in older adults.

**Methods:**

This systematic review and network meta-analysis included a search in PubMed, Embase, and the Cochrane Library for randomized controlled trials from inception to 19 October 2024 of resistance training for patients with senile sarcopenia. Comprehensive data extraction covered dose, resistance training protocol, demographics, and study duration. Systematic review with Bayesian network meta-analysis (NMA) methodology was employed and results were presented as 95% credible intervals (Crl).

**Results:**

A total of 13 studies involving 711 participants (mean age: 68.29 ± 5.30 years; mean BMI: 24.03 ± 3.43; female: 79.5%) were included in this study. The results of our network meta-analysis showed that resistance training variables (e.g., frequency, intensity, period, and training volume) were effective in improving handgrip strength in older patients with sarcopenia. Among them, the effective dose range for frequency of resistance training was 2–5 times/week, resistance training intensity was 30%–75%, resistance training period was 4–24 weeks, resistance training exercise was 3–17 exercises per set, resistance training repetitions was 10–24 reps, resistance training sets was 2–8 sets, resistance training volume was 528–2,200 reps per week. The optimal dose of resistance training to improve handgrip strength in older adults with sarcopenia is 3 times per week (MD = 7.02, 95% CrI [4.62, 9.42]), 49% 1RM (MD = 7.11, 95% CrI [2.69, 11.52]), 19 weeks (MD = 7.87, 95% CrI [3.89, 11.85]), 15 exercises (MD = 8.16, 95% CrI [3.65, 12.66]), 16 reps (MD = 7.62; 95% CrI [4.77, 10.46]), 6 sets (MD = 8.63; 95% CrI [5.06, 12.21]), 1,400 reps/week (MD = 8.45; 95% CrI [5.50, 11.40]).

**Conclusion:**

Resistance training effectively improves handgrip strength in older adults with sarcopenia. A recommended 19-week program includes 3 sessions per week at 49% 1RM, featuring 15 exercises per set, 6 sets, and 16 repetitions per exercise, totaling up to 1,400 reps weekly.

**Systematic Review Registration:**

https://www.crd.york.ac.uk/PROSPERO/recorddashboard.

## 1 Introduction

Sarcopenia is more prevalent in individuals aged 60 years and older. It is defined as an ageing-associated condition characterized by loss of muscle mass and strength (Cruz-Jentoft et al., 2019; Falcon and Harris-Love, 2017), which leads to an increased risk of many outcomes such as falls (Yeung et al., 2019), sarcopenic obesity, physical disability, poor quality of life and death (Goodpaster et al., 2006; Delmonico et al., 2007). A recent systematic review reported that the prevalence of sarcopenia in older adults was 10%–27% based on different diagnostic criteria (Petermann-Rocha et al., 2022), over 50 million people live with sarcopenia around the world, which is predicted to reach 200 million in 2050 with a conservative estimate (Cruz-Jentoft et al., 2010). Older adults with sarcopenia may require greater social and nursing care, which increased social and economic burdens, the prevention of sarcopenia is one of the greatest public health challenges of the 21st century (Fielding et al., 2011; Studenski et al., 2014; Dent et al., 2018).

Decreased muscle strength, as evidenced by low handgrip strength, is one of the main features of sarcopenia in older adults (Chen et al., 2020; Benz et al., 2024). While grip strength is a basic measure of muscle strength and has been widely used for screening and diagnosing sarcopenia (Chen et al., 2020; Fantin et al., 2024). In addition, studies have consistently shown that in patients with sarcopenia comorbid with other conditions, such as cardiovascular disease (Liu et al., 2023), chronic renal disease (Ribeiro et al., 2022), type II diabetes (Chen et al., 2023), knee osteoarthritis (Zhang et al., 2023), and obesity (Fantin et al., 2024), loss of muscle strength as assessed by handgrip strength, has been proposed as a powerful predictor of mortality (Ortega et al., 2012; Lopez-Jaramillo et al., 2014; Al Snih et al., 2002) and has become a new vital sign of health (Vaishya et al., 2024). These studies suggest that muscle strength is a risk factor for sarcopenia and other diseases, and that handgrip strength can be used as a rapid and reliable measure of muscle strength with prognostic value in predicting the risk of death from disease, healthcare practitioners can identify potential health problems in older adults at an early stage and can intervene in a timely manner to improve patient outcomes.

Given the adverse consequences of muscle loss, identifying feasible and cost-effective prevention and treatment strategies is critical to the health and wellbeing of older adults. Resistance training (RT) has long been shown to increase muscle mass, strength, and function in older adults and is superior to other forms of exercise (Marzuca-Nassr et al., 2024; Kim et al., 2023; Grgic et al., 2019; Farup et al., 2012; Yamamoto et al., 2021; Fragala et al., 2019; Yasuda, 2022; Zhao et al., 2022). RT is also associated with many other health benefits, such as reduced all-cause and cancer-related mortality (Saeidifard et al., 2019). The prevalence of various comorbidities such as cardiovascular disease (Paluch et al., 2024), musculoskeletal pain (Pedersen et al., 2013), anxiety and depressive symptoms (O'sullivan et al., 2023), and hypertension (Edwards et al., 2023). Moreover, RT benefits bone health by reducing the risk of osteoporosis and fractures and associated morbidity and mortality (Hinman et al., 2023), and improving mitochondrial content and function in skeletal muscle (Groennebaek and Vissing, 2017). Not surprisingly, physical activity guidelines and exercise recommendations often incorporated RT as cornerstone strategies for the prevention and treatment of sarcopenia. Despite established benefits of RT, only 10%–30% of adults report meeting the recommendations of global physical activity guidelines (Bennie et al., 2016). This is largely due to patients not being offered a routine RT program (Offord et al., 2019) and there is considerable variation in those that are delivered in clinical practice (Witham et al., 2020; Talar et al., 2021), with older adults limiting their own participation in resistance training for safety concerns among others. Thus dose-response analysis can determine the optimal dose range for resistance training for older patients with sarcopenia, providing them with a safe and effective resistance training program to expand their physical participation and health benefits.

A latest network meta-analysis summarizing 42 randomized controlled trials (RCTs) involving 3,728 participants revealed that RT was the one of most effective interventions to improve handgrip strength (MD: 2.69 kg, 95% CI: 1.78–3.61) (Shen et al., 2023). However, the optimal resistance training exercise prescription for improving grip strength in older adults with sarcopenia remains undetermined. It is also unclear what dose response exists between handgrip strength and RT variables in older adults with sarcopenia. This is mainly due to the heterogeneity of the research, the limited nature of the research methodology. Network meta-analysis, also known as mixed treatment comparison or multiple treatment comparison meta-analysis, provides a way to compare and rank the effect sizes of different exercise doses on sarcopenia by estimating direct and indirect comparisons (Bafeta et al., 2014) and can be overcome these limitations.

This study explored the dose-response relationship between several RT manipulable variables (e.g., frequency, intensity, periodization, exercises, sets, repetitions, and RT volume) for older adults with sarcopenia and grip strength using a model-based meta-analytic analysis of dose-response networks in a Bayesian framework and drawing on existing RCT data. We hypothesize that there exists an optimal window for each training variable that maximizes handgrip strength gains. This study provides an evidence-based basis for the construction of a resistance training program for handgrip strength enhancement in older adults with sarcopenia, as well as for clinical practice. At the same time, this research will enable exercise prescription guidelines to be made more specific and scientific and to better meet individual needs, thus playing a greater role in health management and disease prevention.

## 2 Methods

This pre-registered systematic review with meta-analysis (PROSPERO reference number #CRD4202024618543) was reported following the PRISMA checklist (Page et al., 2021; Moher et al., 2009).

### 2.1 Search strategy

We conducted a systematic search in PubMed, Embase and Cochrane Central Register of Controlled Trials (CENTRAL) from inception to 19 October 2024, and including search terms, dates, and process, are shown in Supplementary Material S1. The reference lists of relevant articles and reviews were also screened for additional studies. Title/abstract and full-text screening were conducted independently and in duplicate by investigators (LHR and TSH), with disagreements resolved by discussion or adjudication by a third author (JN) (Nasser, 2020).

### 2.2 Selection criteria

We included (1) randomized controlled trials that (2) individuals were assessed with data available on sarcopenia, severe sarcopenia, or other combinations of physical capability markers called sarcopenia, and (3) used Resistance Training as intervention, (4) the control group (CON) included non-intervention, regular daily activities, health education or usual care. (5) To be considered, had to include the outcome measures of muscle strength: handgrip strength. We excluded studies that (1) non-English publications; and (2) not clearly describe the dose of resistance training; and (3) duplicated publications.

### 2.3 Data extraction

Two authors independently extracted data from studies that met the inclusion criteria (LHR and HSL) and disagreements were resolved by consensus between all authors. From each of the included studies, we extracted relevant publication information (i.e., author and year), number of patients, patient characteristics (e.g., age and sex), interventions considered and outcome measures (handgrip strength). In the process of extracting data, if the original study reported a standard error in the experimental and control groups, the standard deviation was calculated by the formula: In cases where both standard deviation (SD) and other direct measures are unavailable, we will derive the SD using methods based on confidence intervals, quartiles, ranges, or p-values. These methods are outlined in detail in Sect. 7.7.3 of the Cochrane Handbook for Systematic Reviews of Interventions.

### 2.4 Data coding and management

We categorized the interventions into two hierarchical levels: First, interventions were coded as “RT” or “CON” (first level). At second level, the interventions were coded according to resistance training variables: Frequency (number of resistance training sessions completed per week, 2-6 sessions/week), Intensity (training load of a single resistance training session, 30%–75% 1RM), Period (duration of resistance training, 4–24 weeks), Exercises (types of exercises completed per resistance training set, 4–17 exercises), Sets (number of sets completed per resistance training session, 2–10 sets), Repetitions (number of repetitions of individual exercise components, 10–30 reps), and Training volume (total amount of training per week, derived from the product of frequency, exercises, repetitions, and sets, 528–2,448 reps/week), resistance training dose ranges were based on the range of data extracted from the included studies. In order to avoid bias of recorded information caused by subjective factors, the methodology for recording the elements of the exercise dose is described in the Supplementary Material S5 and the final analytical dataset is shown in Supplementary Material S6.

### 2.5 Data synthesis

We used a random-effects Bayesian Model-Based Network Meta-Analysis (MBNMA) (Mawdsley et al., 2016) to summarize the dose-response association between exercise dose and muscle strength with sarcopenia patients based on the R statistical environment (V.4.4.1, https://www.r-project.org). Connectivity between different intervention studies was assessed through a network plot analysis, with nodes representing interventions and connecting lines representing direct comparisons between two interventions. Transitivity was evaluated using node-splitting analysis comparing the differences between direct and indirect estimates for each comparison. Consistency is assessed through the UME model, which assesses whether direct and circumstantial evidence are consistent with each other. No indication of violation of key assumptions for network meta-analysis (i.e., connectedness of the network (Ter et al., 2019), consistency in the data, and transitivity (Wheeler et al., 2010; White et al., 2012) was found (Supplementary Material S3). In order to assess the robustness of the findings, we performed model fitting, as analyzed in Supplementary Material S4 (e.g., density plot, split chart). All effect sizes were reported as mean differences (MD), and 95% credible intervals (CrI) were used to assess the credibility of our estimates (Etzioni and Kadane, 1995).

## 3 Results

### 3.1 Characteristics of included studies

**FIGURE 1:**
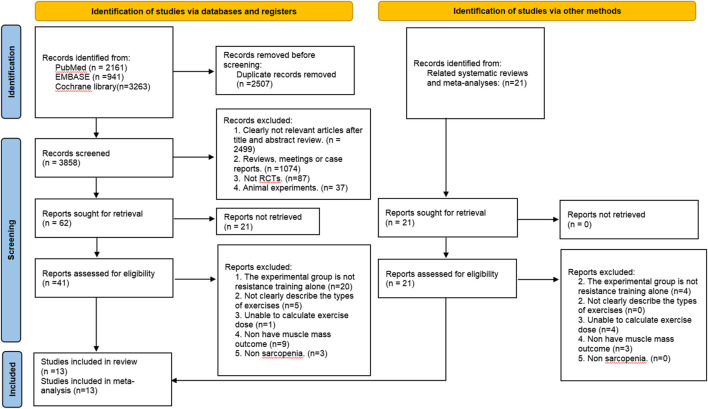
PRISMA Flow diagram of the search process for studies.

Overall, 6,365 records were identified through the initial electronic searches. After removing duplicates, 3,858 records were screened for titles and abstracts and 62 full-text articles were screened for eligibility. A total of 21 records that may have been missed during the search were obtained by reviewing recent reviews or similar meta-analyses related to the search topic and 21 full-text articles were screened for eligibility. In total, 13 studies involving 711 participants (431 treatments and 280 controls) were included in the review (Figure 1). The mean age was 68.29 ± 5.30 years, mean BMI was 24.03 ± 3.43 and female proportion was 79.5%.

The characteristics of included studies are shown in Table 1. The year of publication ranged from 2016 to 2023. We also reported the tools used to assess sarcopenia in the included studies and their cut-offs for sarcopenia Among them, diagnosis of Asian Working Group for Sarcopenia (AWGS) was the most used (5 studies). European Working Group on Sarcopenia in Older People (EWGSOP, 4 studies), European Consensus on Sarcopenia (ECS, 1 study) and three used unspecified regional criteria.

**TABLE 1:** Basic Characteristics of included studies.

Study ID	Age (Mean ± SD)	Sample size (M/F)	Muscle mass	BMI	Diagnosis of sarcopenia	Intervention detail
Silva et al. (2024)	RT: 63.0 ± 6.6 CON: 69.5 ± 5.7	RT: 39 (12/27) CON: 32 (6/26)	SMI RT: 8.7 ± 1.6 CON: 8.1 ± 1.8	RT: 28.0 ± 4.8 CON: 27.8 ± 5.4	ECS HG < 27 kg for men, <16 kg for women; MMI<10.76 kg/m^2^ for men, <6.76 kg/m^2^ for women MMI: RT: 8.7 ± 1.6; CON: 8.1 ± 1.8	RT: 1 h, based on machine or barbell, 3 times/week, 11 exercises, 6–15 reps/set, 60%–85% 1 RM
Xiao et al. (2023)	RT: 53.9 ± 14.5 CON: 51.2 ± 12.9	RT: 30 (13/17) CON: 30 (17/13)	SMM RT: 22.7 ± 7.0 CON: 24.1 ± 8.6	RT: 17.2 ± 2.2 CON: 17.0 ± 2.3	AWGS ① ASMI<7.0 kg/m^2^ in men,<5.7 kg/m^2^ in women; HG < 28 kg in men and <18 kg in women; or speed<1.0 m/s HG: RT: 18.5 ± 4.9; CON: 19.1 ± 5.9 ASMI: RT: 5.42 ± 0.73; CON: 5.64 ± 0.81	RT: based on resistance band, 6 times/week, 4 exercises, 30 reps/set, 3 set/day, 6–9 OMNI
Chen et al. (2018)	RT: 66.7 ± 5.3 CON: 68.3 ± 2.8	RT: 17 (0/17) CON: 16 (0/16)	SMM RT: 20.99 ± 3.00 CON: 19.99 ± 2.74	NA	AWGS ASMI<5.7 kg/m^2^, HG < 18 kg. ASMI: RT: 5.57 ± 0.28; CON: 5.45 ± 0.29 HG: RT: 17.51 ± 3.80; CON: 17.93 ± 4.82	RT: 60 min, based on kettlebell, 2 times/week, 11 exercises, 8–12 reps/set, 3 sets, 60%–70% 1 RM, 2–3 rests
Lee et al. (2021)	RT: 70.13 ± 4.41 CON: 71.82 ± 5.23	RT: 15 (0/15) CON: 12 (0/12)	SMI RT: 5.01 ± 0.83 CON: 5.12 ± 1.0	RT: 26.95 ± 3.31 CON:28.93 ± 3.55	AMI<5.67 kg/m^2^ and HG < 20 kg or speed <0.8 m/s GS: RT: 0.82 ± 0.21; CON: 0.90 ± 0.21 HG: RT: 20.40 ± 4.00; CON: 19.34 ± 6.36	RT: 40 min, based on elastic band, 3 times/week, 5 parts major muscle, 10 reps/set, 3 sets, 13 RPE
Vikberg et al. (2018)	RT: 70.9 ± 0.28 CON: 70.0 ± 0.29	RT: 36 (16/20) CON: 34 (16/18)	AMI RT: 6.25 ± 0.86 CON: 6.24 ± 0.85	RT: 22.72 ± 2.35 CON: 23.33 ± 3.01	EWGSOP AMI ≤7.29 (range: 5.69–7.29) among men, ≤5.93 (range: 4.50–5.93) among women	RT: 45 min, based on one’s body weight, 8 exercises, 3 times/week, 10–12 reps/set, 2–4 sets, CR-10 6–7
Dong et al. (2019)	RT: 59.0 (32.5,66.5) CON: 62.5 (50.5,70.0)	RT: 21 (9/12) CON: 20 (12/8)	SMM RT: 21.19 ± 3.65 CON: 1.06 ± 3.12	RT: 18.96 ± 3.08 CON: 20.49 ± 3.41	AWGS SMI: male <7.0 kg/m^2^, female <5.7 kg/m^2^; HG: (male <26 kg, female <18 kg), speed (<0.8 m/s) SMI: RT: 5.70 ± 0.80; CON: 5.87 ± 0.69	RT: 1–2 h, based on own body weight and elastic balls, 3 times/week, 10 reps/set, 10 sets, 0.5 kg/week, 0–5 kg weight
Yamada et al. (2019)	RT: 84.7 ± 5.1 CON: 83.9 ± 5.7	RT: 28 (10/18) CON: 28 (13/15)	AMM RT: 17.19 ± 4.58 CON: 17.63 ± 5.11	RT: 22.6 ± 3.0 CON: 21.2 ± 2.9	AWGS	RT: 20 min, based on body weight or elastic band, 2 times/week, 7 exercises, 20 reps/set, 3 sets
Gadelha et al. (2021)	RT: 65.0 ± 3.6 CON: 63.8 ± 4.1	RT: 37 (−/−) CON: 28 (−/−)	NA	RT: 20.8 ± 5.7 CON: 20.7 ± 4.7	EWGSOP-2 FFMI <15.3 and 15.6 kg/m^2^ for men and women; handgrip strength<30 kg and 20 kg for men and women HG: RT: 23.1 ± 2.2; CON: 25.1 ± 3.0	RT: 40 min, based on one’s body weight, 6 exercises, 8–12 reps/set, 3 sets, 5–8 OMNI, 2 min rest
Seo et al. (2021)	RT: 70.3 ± 5.38 CON: 72.9 ± 4.75	RT: 12 (0/12) CON: 10 (0/10)	AMM RT: 12.3 ± 0.96 CON: 12.4 ± 0.95	RT: 22.9 ± 2.02 CON: 22.4 ± 1.52	EWGSOP (1) GS < 1.0 m·s^−1^ and ASMI <5.67 kg·m^−2^, or GS > 1.0 m·s^−1^, (2) grip strength<20 kg, ASMI<5.67 kg·m^−2^ (3) PBF< 35%; (4) T-score < −2.5	RT: 50 min, based on elastic band, 17 exercises, 3 times/week, 6–15 reps, 3–5 sets, 4–8 OMNI Scale, 1 min rest
Chien et al. (2022)	RT: 67.6 ± 7.7 CON: 67.3 ± 6.1	RT: 20 (5/15) CON: 20 (2/18)	AMI RT: 6.9 ± 0.9 CON: 6.9 ± 0.7	RT: 24.3 ± 3.4 CON: 25.5 ± 3.7	AWGS: CC < 34 cm, GS < 28 kg for males. CC < 33 cm and GS < 18 kg for females CC: RT: 30.4 ± 2.3; CON: 31.3 ± 2.0 GS: RT: 15.9 ± 5.0; CON: 15.1 ± 3.8	RT: 30 min, based on Sandbags, 5 exercises, 3 times/week, 8–15 reps, 3 sets, 1–2 min rest, 13 RPE
de Sá Souza et al. (2022)	RT: 77.42 ± 6.25 CON: 74.64 ± 7.13	RT: 14 (7/7) CON: 14 (3/11)	AMI (man) RT: 7.51 ± 0.48 CON: 7.13 ± 0.44 AMI (woman) RT: 5.85 ± 0.57 CON: 6.28 ± 0.73	RT: 25.54 ± 2.04 CON: 26.78 ± 4.44	AMI<7.27 g/m^2^、HG < 40 kg、SPPB-score <6 points in man, AMI<5 g/m^2^, HG < 30 kg, SPPB score<6 points in women SPPB (men) RT: 10.0 ± 1.15; CON: 10.0 ± 1.73 SPPB (women) RT: 5.85 ± 0.57; CON: 8.63 ± 2.73 HG (men) RT: 26.41 ± 5.10; CON: 27.66 ± 4.93 HG (women) RT: 16.57 ± 2.50; CON: 18.82 ± 6.65	RT: based on one’s own weight, 3 times/week, 8 exercises, 2 sets, 10–12 reps, 50%–75% 1 RM
Chen et al. (2017)	RT: 68.9 ± 4.4 CON: 68.6 ± 3.1	RT: 15 (3/12) CON: 15 (2/13)	SMM RT: 22.9 ± 4.0 CON: 21.6 ± 3.6	RT: 28.3 ± 4.4 CON: 29.0 ± 3.9	ASM (kg)/Weight (kg)*100%: men ≤32.5%; women ≤25.7% RT: 24.1 ± 2.4; CON: 23.0 ± 2.6	RT: 2 times/week, 10 exercises, 60%–70% 1 RM, 8–12 reps/set, 3 sets, 2–3 min rest, from seated to standing exercises
Liao et al. (2017)	RT: 66.39 ± 4.49 CON: 68.42 ± 5.86	RT: 25 (0/25) CON: 21 (0/21)	SMI RT: 6.85 ± 0.33 CON: 6.91 ± 0.24	RT: 27.32 ± 3.33 CON: 28.19 ± 3.27	EWGS SMI ≤7.15 kg/m^2^ RT: 6.85 ± 0.33; CON: 6.91 ± 0.24	RT: 35–40 min, based on the band, 6 exercises, 10–20 reps/set, 3 sets, 10–13 RPE

Note. M/F: male or female; RT: resistance training; CON: control group; SMI: Skeletal muscle mass index = SMM/height2; SMM: skeletal muscle mass; ASM: appendicular skeletal muscle mass, ASI = ASM/height^2^ = ASMI; ECS: european consensus on sarcopenia; EWGSOP: European Working Group on Sarcopenia in Older People = EWGS; AWGS: asian working group for sarcopenia; CC: calf circumference; SPPB: short physical performance battery; GS: gait speed. PBF: percentage body fat; BMI: body mass index; NA: not available.

### 3.2 Dose-response relationships

#### 3.2.1 Effect of frequency on handgrip strength improvement in older adults with sarcopenia

**FIGURE 2:**
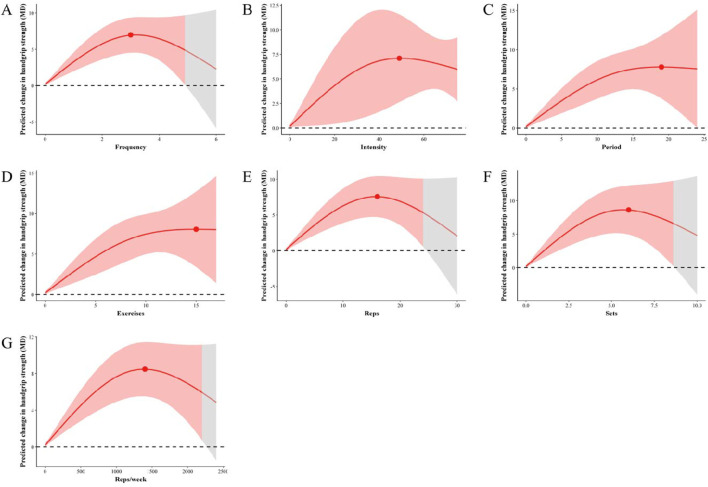
Dose-response relationship using a restricted cubic spline. Red lines represent the estimated hazard ratio, the shaded red area corresponds to the confidence intervals. **(A)** Association between resistance training frequency and handgrip strength in older adults with sarcopenia **(B)** Association between resistance training intensity and handgrip strength in older adults with sarcopenia **(C)** Association between resistance training period and handgrip strength in older adults with sarcopenia **(D)** Association between resistance training exercises and handgrip strength in older adults with sarcopenia **(E)** Association between resistance training reps and handgrip strength in older adults with sarcopenia **(F)** Association between resistance training sets and handgrip strength in older adults with sarcopenia **(G)** Association between resistance training reps/week and handgrip strength in older adults with sarcopenia. The red shaded areas represent the significantly effective, and the red points represents the optimal effect dose.

We employed restricted cubic spline (RCS) functions, as depicted in Figure 2A, to investigate potential nonlinearity in the relationships between resistance training frequency and handgrip strength in old adults with sarcopenia. We found that the frequency of resistance training was associated with improved handgrip strength in older adults with sarcopenia, and observed an inverted U-shaped curve for the relationship between resistance training frequency and handgrip strength in older adults with sarcopenia. Predicted maximal significant response was observed at 3 times/week (MD = 7.02, 95% CrI [4.62, 9.42]). In addition, 2–5 times/week was the effective dose range in which resistance training appears to significant enhance handgrip strength in old adults with sarcopenia.

#### 3.2.2 Effect of intensity on handgrip strength improvement in old adults with sarcopenia

Figure 2B illustrates the dose-response relationships of resistance training intensity and handgrip strength in old adults with sarcopenia based on RCSs. The RCS analysis revealed an inverted U-shaped curve for the relationship between resistance training intensity and handgrip strength in older adults with sarcopenia. Predicted maximal significant response was observed at 49% 1RM (MD = 6.97, 95% CrI [2.65, 11.21]). Our results showed that 30%–75% 1RM was an effective intensity dose range for resistance training to significantly enhance handgrip strength in older adults with sarcopenia.

#### 3.2.3 Effect of periodization on handgrip strength improvement in old adults with sarcopenia

We utilized RCS to analyze and illustrate the nonlinear associations of resistance training period and handgrip strength in older adults with sarcopenia (Figure 2C). Our study demonstrated that resistance training period was associated with improved handgrip strength in older adults with sarcopenia, The RCS demonstrated an inverted U-shaped curve for the relationship between resistance training period and handgrip strength in older adults with sarcopenia. Predicted maximal significant response was observed at 19 weeks (MD = 7.87, 95% CrI [3.89, 11.85]). We detected that 4–24 weeks was the effective dose range for resistance training to significant enhance handgrip strength in old adults with sarcopenia.

#### 3.2.4 Effect of exercises on improvement of handgrip strength in older adults with sarcopenia

We further assessed the dose-response relationship resistance training exercises and handgrip strength in old adults with sarcopenia using RCSs. Our study showed an inverted U-shaped curve for the relationship between resistance training exercises and handgrip strength in older adults with sarcopenia. Predicted maximal significant response was observed at 15 exercises (MD = 8.16, 95% CrI [3.65, 12.66]). Figure 2D showed that 3–17 exercises was the statistically significant resistance training exercises range dose to enhance handgrip strength in old adults with sarcopenia.

#### 3.2.5 Effect of reps on handgrip strength improvement in older adults with sarcopenia

To analyze the dose-response relationships between resistance training reps and handgrip strength in old adults with sarcopenia, a RCS model was used (Figure 2E). The RCS demonstrated an inverted U-shaped curve for the relationship between resistance training repetitions and handgrip strength. Predicted maximal significant response was observed at 16 reps (MD = 7.62; 95% CrI [4.77, 10.46]). Resistance training reps presented 10–24 reps was the effective dose range that achieve to improve handgrip strength in older patients with sarcopenia.

#### 3.2.6 Effect of the sets on handgrip strength improvement in older adults with sarcopenia

The RCS model was established to explore the nonlinear relationship between resistance training sets and handgrip strength in old adults with sarcopenia. The results showed a strong nonlinear association between resistance training sets and handgrip strength, and the dose-response curve exhibits an inverted U shape (Figure 2F). Predicted maximal significant response was observed at 6 sets (MD = 8.63; 95% CrI [5.06, 12.21]). We detected that 2-8 sets was the effective dose range in which resistance training appears to significant enhance handgrip strength in old adults with sarcopenia.

#### 3.2.7 Effect of training volume on handgrip strength improvement in older adults with sarcopenia

In Figure 2G we used RCS to flexibly model predicted the dose-response relationships of reps/week and handgrip strength in old adults with sarcopenia. RCS analysis indicated an inverted “U-shaped” association between resistance training volume and handgrip strength in old adults with sarcopenia. Predicted maximal significant response was observed at 1,400 reps/week (MD = 8.45; 95% CrI [5.50, 11.40]). A significant effect of resistance training reps/week on handgrip strength in older adults with sarcopenia were found that 528–2,200 reps/week was the statistically significant resistance training volume range dose.

## 4 Discussion

To the best of our knowledge, this is the first comprehensive dose-response meta-analysis that provided the evidence of the relationship between RT exercise doses and handgrip strength in sarcopenia patients with ultimately included 13 eligible randomized controlled trials involving 711 participants. The current study provides several key findings that is important for clinicians. First, we performed a dose-response meta-analysis to determine how much RT exercise doses modify the RT exercise effects on handgrip strength in older adults with sarcopenia, a non-linear relationship was observed between RT exercise doses and handgrip strength with an inverted U-shaped curve. Second, this dose-response meta-analysis showed that the effective dose range for frequency of resistance training was 2–5 times/week, resistance training intensity was 30%–75%, resistance training period was 4–24 weeks, resistance training exercises was 3–17 times per week, resistance training repetitions was 10–24 reps, resistance training sets was 2–8 sets, resistance training volume was 528–2,200 reps per week. Third, the optimal dose of resistance training to improve handgrip strength in older adults with sarcopenia is 3 times per week, 49% 1RM, 19 weeks, 15 exercises, 16 reps, 6 sets, 1,400 reps/week (Table 2). In summary, our findings provide an opportunity to inform future exercise guidelines aimed to improve handgrip strength in older adults and reduce the burden associated with sarcopenia in this growing population.

**TABLE 2:** Statistical parameters for different training variables.

Resistance training variables	Range of dose	Statistically significant dose ranges	Optimal dose	MD	95%CrI
Frequency	2–6 times/week	2–5 times/week	3 times/week	8.16	[3.65, 12.66]
Intensity	30%–75% 1RM	30%–75% 1RM	49% 1RM	6.97	[2.65, 11.21]
Periodization	4–24 weeks	4–24 weeks	19 weeks	7.87	[3.89, 11.85]
Exercises	3–17 exercises per set	3–17 exercises per set	15 exercises	8.16	[3.65, 12.66]
Reps	10–30 reps	10–24 reps	16 reps	7.62	[4.77, 10.46]
Sets	3–10 sets	2–8 sets	6 sets	8.63	[5.06, 12.21]
Training volume	528–2,448 reps per week	528–2,200 reps per week	1,400 reps/week	8.45	[5.50, 11.40]

RT frequency is recognized as an important variable in the muscle growth response induced by regular resistance training. In the absence of considering exercise dosage, our research findings indicated that a training frequency of 2–5 times per week had a statistically meaningful frequency dosage with the optimal dose (3 times/week) for enhancing handgrip strength in older adults with sarcopenia. Firstly, the time course of muscle protein synthesis (MPS) diminishes with individual resistance training experience, higher training frequency leads to greater muscle strength gains (Schoenfeld et al., 2016; Damas et al., 2015). It is worth noting that large training volumes associated with excessive training frequency lead to cumulative fatigue, which ultimately affects athletic performance (Ribeiro et al., 2015; Chilibeck et al., 1998). However, proper recovery time can aid in muscle growth and strength gains (American College of Sports Medicine, 2009). Particularly, the harder a muscle group trains, the longer it takes to recover (American College of Sports Medicine, 2009; Hackett et al., 2013). Therefore, resistance training 3 times a week ensures adequate recovery time for the muscles while maintaining a high training frequency to promote muscle adaptation and growth. Although the optimal RT frequency to elicit adaptive changes in skeletal muscle may be influenced by multiple factors, training each muscle group at a frequency of 3 times per week provides optimal stimulation to maximize strength in older adults. Uncertainty due to the small number of studies addressing frequency parameters requires more standardized intervention reports to guide future research.

Resistance training intensity is arguably the most important exercise dose for stimulating muscle growth, usually defined as the individual percentage of 1RM. Our study provided a statistically significant range of intensity doses (30%–75% 1RM) for enhancing handgrip strength in older adults with a plateau in the handgrip strength enhancement effect occurred at 49% 1RM. Previous studies have shown that high intensity RT has the greatest impact on muscular strength compared to moderate- or low-intensity training (Beneka et al., 2005; Vincent et al., 2002; Hortobagyi et al., 2001). Similar results were not observed in our study. Firstly, it becomes progressively more difficult to gain lean muscle mass as training experience increases and the body adapts to the intensity of the exercise, which leads to stagnation of the training effect (Schoenfeld, 2010). It should be pointed out that the repetitions is often used as an indicator of training intensity, and the individual percentage of 1RM determines the realized number of repetitions within a set until failure (Silva et al., 2014). However, some studies have shown that a given number of repetitions cannot be correlated with a specific percentage of 1 RM (Silva et al., 2014; Borde et al., 2015), the individual percentage of 1 RM is a stress rather than a strain factor. Therefore, in order to individualize resistance training, future research should focus on finding an effective strain-based method to efficiently quantify training intensity.

Resistance training period is the cumulative number of weeks of performing the RT program. Our study demonstrated a statistically significant improvement in handgrip strength in older patients with sarcopenia for a duration of 4–24 weeks with a optimal dose-responses at 19 weeks. A meta-regression revealed a dose-response relationship between training period and muscle strength gains (Silva et al., 2014; Borde et al., 2015). Longer training durations had a greater impact on strength gains than shorter training periods over the course of 8–52 weeks of training (Silva et al., 2014), which is contrary to our findings. Previous study reported a simple linear relationship between training period and muscle strength that handgrip strength increases progressively with RT training duration, the complex relationship between training period and muscle strength was not characterized by model fitting and produced untrustworthy results. Additionally, large-volume, high-intensity and long-term resistance training interventions can result in over-training, eliciting adverse physiological responses, which in turn can lead to trainer resistance and affect training adherence. Nevertheless, as the training duration increases, the muscle adapts to the training load and growth slows down. Therefore, appropriate changes to an RT program can avoid training plateaus, maximize muscle strength, and reduce the likelihood of over training.

Resistance training volume represents total amount of training per week, derived from the product of frequency, exercises, repetitions and sets. Our results suggest that the optimal resistance training volume for improving handgrip strength in older adults with sarcopenia is a resistance training program of six sets of 15 exercises with 16 repetitions of each movement three times per week. Firstly, a strong evidence suggests that muscle strength gains are associated with increased training volume (Schoenfeld et al., 2019), and several studies reported that RT volume has an inverted U-shaped response relationship with skeletal muscle strength adaptation (Aube et al., 2022; Figueiredo et al., 2018), which consistent with our findings. Increasing RT volume can be accomplished by increasing the number of sets, frequency, number of exercises, and repetitions. However, low exercise dosages may not be enough to cause an increase in muscle strength (Schoenfeld et al., 2016), whereas excessively high dosages may lead to physical fatigue (Ribeiro et al., 2015; Chilibeck et al., 1998). In general, completing 50%–70% of the maximal repetitions possible performed with good posture is sufficient to elicit neuromuscular improvements while avoiding poor posture and injury (Da Silva et al., 2018). Therefore, health professionals should take this into consideration when designing optimal resistance training programs for older adults with sarcopenia to ensure that each patient benefits from resistance training therapy while reducing potential risks. Nonetheless, more research is warranted to explore which and how these variables can be manipulated to enhance muscle physiological responses in elderly patients with sarcopenia.

Notably, we found in these associations that RT variables were associated with improved handgrip strength in older adults with sarcopenia and detected an invert U pattern. These results are generally consistent with the results of previous studies (Schoenfeld et al., 2016; Grgic et al., 2022; Buckner et al., 2021), and may reflect the different mechanisms and pathways of action for changes in handgrip strength induced by different RT variables. Our finding provides important insights into the prevention and treatment of sarcopenia in older adults and also emphasizes the potential for promoting resistance training as a health promotion measure. However, further research on the effectiveness of resistance training in older adults with different health conditions is needed to fully understand the applicability of different doses of resistance training in the older adults. The low participation rates and broad health benefits underscore the need for evidence-based guidelines and recommendations for resistance exercise for older adults to safely and beneficially incorporate strength training into their lives. We recommend a 19-week program for older adults with sarcopenia that consists of 3 sessions per week at 49% 1 RM, featuring 15 exercises per set, 6 sets, and 16 repetitions per exercise, totaling up to 1,400 reps weekly. However, we also found large differences in the way interventions were reported across studies and a lack of uniform standardized norms. This makes it difficult to synthesize and compare the results of multiple studies. In addition, due to the small number of studies included in this study, the multitude of interventions and complexity of the training variables indirectly influenced the uncertainty of the results of this study, and more standardized intervention reports are needed to guide future research.

This dose-response analysis has several major clinical implications. First, the information we provide can be directly used to recommend what dose of resistance training is recommended for optimal grip strength in older adults. Second, our findings suggest that a dose of exercise is always better than no dose of exercise, which also suggests that the American College of Sports Medicine’s recommends of “resistance training at least twice a week” may be a more feasible and effective recommendation for improving muscle strength in older adults. This provides patients and clinicians with valuable insights highlighting practical resistance training strategies for improving muscle strength in older adults. As a result, clinicians, healthcare professionals, or patients can make individualized choices based on personal preference and physical condition to most effectively improve skeletal muscle mass in older adults.

We need to recognize and consider several limitations of this study. First, the most apparent limitation of the current review is the small number of studies that included trained participants, although we included a considerable sample size of older adults with sarcopenia, only a few eligible studies were included in the analyses for some specific interventions and outcomes. Such an inadequate sample size may have limited the reliability and accuracy of the comparison of statistical results between exercise dose and grip strength improvement. We attempt to mitigate this problem by using a random effects model. Second, it is important to assess the magnitude of the effect of resistance training dose on handgrip strength improvement in patients with sarcopenia. However, due to the complexity and diversity of exercise doses, the interpretation and extrapolation of results are often subject to some uncertainty. Therefore, larger randomized controlled trials are needed to address uncertainty about the optimal dose of resistance training in sarcopenia. Third, despite the coding and management of our data, some of the dosage parameters for resistance training are not clearly documented or poorly articulated in the literature, which may affect the reliability of the results of this study. Finally, due to the diversity of myasthenia gravis assessment tools in this meta-analysis, this may affect the generalizability of our estimates. In the meantime, we should treat the findings of this meta-analysis with caution, and large-scale high-quality studies are still needed for deeper analytical investigations in the future. In summary, our findings mark an important step in providing accurate exercise recommendations for improving muscle strength in older adults.

## 5 Conclusion

This systematic review and meta-analysis identified a dose-response relationship between different variables of resistance training and handgrip strength in older adults with sarcopenia, that confirmed our initial research hypothesis that each training variable has an optimal window for maximizing handgrip strength. Our findings support that resistance training can effectively improves handgrip strength in older adults with sarcopenia. A recommended 19-week program (MD = 7.87, 95% CrI [3.89, 11.85]) includes 3 sessions per week (MD = 7.02, 95% CrI [4.62, 9.42]) at 49% 1RM (MD = 7.11, 95% CrI [2.69, 11.52]), featuring 15 exercises per set (MD = 8.16, 95% CrI [3.65, 12.66]), 6 sets (MD = 8.63; 95% CrI [5.06, 12.21]), and 16 repetitions per exercise (MD = 7.62; 95% CrI [4.77, 10.46]), totaling up to 1,400 reps weekly (MD = 8.45; 95% CrI [5.50, 11.40]). In addition, errors in data recording and fitting models to experimental data can introduce uncertainty to the estimation results. Therefore, this study aimed to establish quantitative analysis evidence of resistance training dose and grip strength enhancement in order to guide elderly patients with sarcopenia on how to scientifically perform resistance training to enhance handgrip strength.

## Data Availability

The original contributions presented in the study are included in the article/Supplementary Material, further inquiries can be directed to the corresponding authors.
