# Supplementary material for: Optimal dose of resistance training to improve handgrip strength in older adults with sarcopenia: a systematic review and Bayesian model-based network meta-analysis
Source: Front Physiol. 2025 Jul 2;16:1564988. doi: 10.3389/fphys.2025.1564988 (PMC12263917; doi:10.3389/fphys.2025.1564988)
Supplement: Supplementary file 1 [file Table1.docx]

**Optimal dose of exercise to improve muscle strength in older adults with sarcopenia: A systematic review and Bayesian model-based network meta-analysis of RCTs**

**Table of Contents**

[Supplementary file 1: Search strategy 2](#_Toc9055)

[Supplementary file 2: Characteristics of included studies 7](#_Toc295)

[Supplementary file 3: Key assumptions of Network Meta-Analysis 10](#_Toc29641)

[Supplementary Figure 1. Schematic diagram of network meta-statistics analysis 11](#_Toc31267)

*[Connectivity](#_Toc1479)* [11](#_Toc1479)

[Supplementary Figure 2. Treatment-level network. 11](#_Toc6775)

*[Consistency](#_Toc14976)* [11](#_Toc14976)

[Supplementary Table 1. Consistent and UME models fit comparison 12](#_Toc28999)

*[Transitivity](#_Toc13503)* [14](#_Toc13503)

[Supplementary Table 2. Node-splitting analysis of inconsistency 14](#_Toc28060)

[Supplementary Figure 3. Node-splitting analysis (density plot). 19](#_Toc18729)

[Supplementary file 4: Non-linear functions and models fit comparison 20](#_Toc13376)

[Supplementary Figure 4. “Split” NMA of different Exercise dose. 20](#_Toc2356)

[Supplementary Table 3. Models fit comparison 21](#_Toc22879)

[Supplementary Figure 5. The deviance plot for treatment effects. 28](#_Toc1724)

[Supplementary file 5: Characteristics of included studies 29](#_Toc17466)

[Supplementary Table 4. Methods of data coding and management 29](#_Toc7535)

[Supplementary file 6: Characteristics of included studies 31](#_Toc30069)

[List of included studies 33](#_Toc14436)

[References 34](#_Toc27842)

# Supplementary file 1: Search strategy

| PubMed  (2024-10-19) | 1. Sarcopenia[MeSH Terms] 2. ("Sarcopenia"[Title/Abstract]) OR ("Sarcopenias"[Title/Abstract]) 3. (muscle[Title/Abstract]) OR (muscular[Title/Abstract]) 4. ((((atroph*[Title/Abstract]) OR (wasting*[Title/Abstract])) OR (weak*[Title/Abstract])) OR (loss*[Title/Abstract])) OR (depletion*[Title/Abstract]) 5. ((muscle[Title/Abstract]) OR (muscular[Title/Abstract])) AND (((((atroph*[Title/Abstract]) OR (wasting*[Title/Abstract])) OR (weak*[Title/Abstract])) OR (loss*[Title/Abstract])) OR (depletion*[Title/Abstract])) 6. ((Sarcopenia[MeSH Terms]) OR (("Sarcopenia"[Title/Abstract]) OR ("Sarcopenias"[Title/Abstract]))) OR (((muscle[Title/Abstract]) OR (muscular[Title/Abstract])) AND (((((atroph*[Title/Abstract]) OR (wasting*[Title/Abstract])) OR (weak*[Title/Abstract])) OR (loss*[Title/Abstract])) OR (depletion*[Title/Abstract]))) 7. Aged[MeSH Terms] 8. (((((("Elderly"[Title/Abstract]) OR ("senior citizen "[Title/Abstract])) OR ("elderly people"[Title/Abstract])) OR ("elderly patients"[Title/Abstract])) OR ("old people"[Title/Abstract])) OR ("senile"[Title/Abstract])) OR ("aged people"[Title/Abstract]) 9. (((old[Title/Abstract]) OR (elder[Title/Abstract])) OR (aged[Title/Abstract])) AND (adult[Title/Abstract]) 10. ((Aged[MeSH Terms]) OR ((((((("Elderly"[Title/Abstract]) OR ("senior citizen "[Title/Abstract])) OR ("elderly people"[Title/Abstract])) OR ("elderly patients"[Title/Abstract])) OR ("old people"[Title/Abstract])) OR ("senile"[Title/Abstract])) OR ("aged people"[Title/Abstract]))) OR ((((old[Title/Abstract]) OR (elder[Title/Abstract])) OR (aged[Title/Abstract])) AND (adult[Title/Abstract]))Resistance Training/ 11. Exercise[MeSH Terms] 12. sports[MeSH Terms] 13. "Exercise Therapy"[MeSH Terms] 14. (sports[MeSH Terms]) OR (sport[Title/Abstract]) 15. (((((((((("Exercise Therapies"[Title/Abstract]) OR ("aerobic exercise*"[Title/Abstract])) OR ("Physical Endurance"[Title/Abstract])) OR ("balance training"[Title/Abstract])) OR ("mixed exercise training"[Title/Abstract])) OR ("combined exercise training"[Title/Abstract])) OR ("Physical Exercise*"[Title/Abstract])) OR (physical* activ*[Title/Abstract])) OR ("Resistance Training"[Title/Abstract])) OR ("Muscle Stretching Exercises"[Title/Abstract])) OR ("Strength Training"[Title/Abstract]) 16. (((((((((((("Tai Ji"[Title/Abstract]) OR ("Tai-ji "[Title/Abstract])) OR ("Tai Chi"[Title/Abstract])) OR ("Tai Ji Quan"[Title/Abstract])) OR (jogging[Title/Abstract])) OR ("baduanjin"[Title/Abstract])) OR ("Eight-section Brocade"[Title/Abstract])) OR (running[Title/Abstract])) OR (swimming[Title/Abstract])) OR (dance[Title/Abstract])) OR (yoga[Title/Abstract])) OR (training[Title/Abstract])) OR (riding[Title/Abstract]) 17. (randomized controlled trial[pt] OR controlled clinical trial[pt] OR randomized[tiab] OR placebo[tiab] OR drug therapy[sh] OR randomly[tiab] OR trial[tiab] OR groups[tiab]) NOT (animals[mh] NOT humans[mh]) 18. (randomized controlled trial[pt] OR controlled clinical trial[pt] OR randomized[tiab] OR placebo[tiab] OR drug therapy[sh] OR randomly[tiab] OR trial[tiab] OR groups[tiab]) NOT (animals[mh] NOT humans[mh]) 19. ((((randomized controlled trial[pt] OR controlled clinical trial[pt] OR randomized[tiab] OR placebo[tiab] OR drug therapy[sh] OR randomly[tiab] OR trial[tiab] OR groups[tiab]) NOT (animals[mh] NOT humans[mh])) AND ((((((Exercise[MeSH Terms]) OR (sports[MeSH Terms])) OR ("Exercise Therapy"[MeSH Terms])) OR ((sports[MeSH Terms]) OR (sport[Title/Abstract]))) OR ((((((((((("Exercise Therapies"[Title/Abstract]) OR ("aerobic exercise*"[Title/Abstract])) OR ("Physical Endurance"[Title/Abstract])) OR ("balance training"[Title/Abstract])) OR ("mixed exercise training"[Title/Abstract])) OR ("combined exercise training"[Title/Abstract])) OR ("Physical Exercise*"[Title/Abstract])) OR (physical* activ*[Title/Abstract])) OR ("Resistance Training"[Title/Abstract])) OR ("Muscle Stretching Exercises"[Title/Abstract])) OR ("Strength Training"[Title/Abstract]))) OR ((((((((((((("Tai Ji"[Title/Abstract]) OR ("Tai-ji "[Title/Abstract])) OR ("Tai Chi"[Title/Abstract])) OR ("Tai Ji Quan"[Title/Abstract])) OR (jogging[Title/Abstract])) OR ("baduanjin"[Title/Abstract])) OR ("Eight-section Brocade"[Title/Abstract])) OR (running[Title/Abstract])) OR (swimming[Title/Abstract])) OR (dance[Title/Abstract])) OR (yoga[Title/Abstract])) OR (training[Title/Abstract])) OR (riding[Title/Abstract])))) AND (((Aged[MeSH Terms]) OR ((((((("Elderly"[Title/Abstract]) OR ("senior citizen "[Title/Abstract])) OR ("elderly people"[Title/Abstract])) OR ("elderly patients"[Title/Abstract])) OR ("old people"[Title/Abstract])) OR ("senile"[Title/Abstract])) OR ("aged people"[Title/Abstract]))) OR ((((old[Title/Abstract]) OR (elder[Title/Abstract])) OR (aged[Title/Abstract])) AND (adult[Title/Abstract])))) AND (((Sarcopenia[MeSH Terms]) OR (("Sarcopenia"[Title/Abstract]) OR ("Sarcopenias"[Title/Abstract]))) OR (((muscle[Title/Abstract]) OR (muscular[Title/Abstract])) AND (((((atroph*[Title/Abstract]) OR (wasting*[Title/Abstract])) OR (weak*[Title/Abstract])) OR (loss*[Title/Abstract])) OR (depletion*[Title/Abstract])))) |
| --- | --- |
| Embase (2024-10-19) | 1. sarcopenia'/exp 2. 'sarcopenia':ab,ti OR 'sarcopenias':ab,ti 3. 'muscle':ab,ti OR 'muscular':ab,ti 4. 'atroph*':ab,ti OR 'wasting*':ab,ti OR 'weak*':ab,ti OR 'loss*':ab,ti OR 'depletion*':ab,ti 5. #3 AND #4 6. #1 OR #2 OR #5 7. 'aged'/exp   'elderly':ab,ti OR 'senior citizen':ab,ti OR 'elderly people':ab,ti OR 'elderly patients':ab,ti OR 'old people':ab,ti OR 'aged people':ab,ti OR 'senile':ab,ti   1. 'exercise'/exp 2. #7 OR #8 3. 'sport'/exp 4. 'kinesiotherapy'/exp 5. 'aerobic exercise*':ab,ti OR 'physical endurance':ab,ti OR 'balance training':ab,ti OR 'mixed exercise training':ab,ti OR 'combined exercise training':ab,ti OR 'physical exercise*':ab,ti OR 'physical* activ*':ab,ti OR 'resistance training':ab,ti OR 'muscle stretching exercises':ab,ti OR 'strength training':ab,ti 6. 'tai chi':ab,ti OR 'jogging':ab,ti OR 'baduanjin':ab,ti OR 'running':ab,ti OR 'swimming':ab,ti OR 'yoga':ab,ti OR 'dancing':ab,ti OR 'resistance training':ab,ti OR 'training':ab,ti 7. #9 OR #11 OR #12 OR #13 OR #14 8. ('crossover procedure':de OR 'double-blind procedure':de OR 'randomized controlled trial':de) AND or AND 'single-blind procedure':de OR (random*:de,ab,ti AND or:de,ab,ti AND factorial*:de,ab,ti) OR crossover*:de,ab,ti OR ((cross NEXT/1 over*):de,ab,ti) OR placebo*:de,ab,ti OR ((doubl* NEAR/1 blind*):de,ab,ti) OR ((singl* NEAR/1 blind*):de,ab,ti) OR assign*:de,ab,ti OR allocat*:de,ab,ti OR volunteer*:de,ab,ti 9. #6 AND #10 AND #15 AND 16 |
| Cochrane (2024-10-19) | 1. #1 MeSH descriptor: [Sarcopenia] explode all trees 884 2. #2 ("sarcopenia"):ti,ab,kw OR ("Sarcopenias"):ti,ab,kw (Word variations have been searched) 2426 3. #3 ("muscle"):ti,ab,kw OR ("muscular"):ti,ab,kw (Word variations have been searched) 108790 4. #4 (atroph*):ti,ab,kw OR (wasting*):ti,ab,kw OR (weak):ti,ab,kw OR (loss*):ti,ab,kw OR (depletion*):ti,ab,kw (Word variations have been searched) 132835 5. #5 #3 AND #4 15219 6. #6 #1 OR #2 OR #5 16858 7. #7 MeSH descriptor: [Aged] explode all trees 273978 8. #8 ("Elderly"):ti,ab,kw OR ("senior citizen"):ti,ab,kw OR ("elderly people"):ti,ab,kw OR ("elderly patients"):ti,ab,kw OR ("old people"):ti,ab,kw (Word variations have been searched) 62230 9. #9 ("senile"):ti,ab,kw OR ("aged people"):ti,ab,kw (Word variations have been searched) 2401 10. #10 (old):ti,ab,kw OR (elder):ti,ab,kw OR (aged):ti,ab,kw (Word variations have been searched) 958762 11. #11 (adult):ti,ab,kw (Word variations have been searched) 860949 12. #12 #10 AND #11 535147 13. #13 #7 OR #8 OR #9 OR #12 641306 14. #14 MeSH descriptor: [Exercise] explode all trees 38461 15. #15 MeSH descriptor: [Sports] explode all trees 21975 16. #16 MeSH descriptor: [Exercise Therapy] explode all trees 21542 17. #17 ("aerobic exercise"):ti,ab,kw OR ("Exercise Therapies"):ti,ab,kw OR ("Physical Endurance"):ti,ab,kw OR ("balance training"):ti,ab,kw OR ("mixed exercise training"):ti,ab,kw (Word variations have been searched) 32996 18. #18 ("combined exercise training"):ti,ab,kw OR ("Exercise Therapies"):ti,ab,kw OR ("Physical Endurance"):ti,ab,kw OR ("balance training"):ti,ab,kw OR ("physical activities"):ti,ab,kw (Word variations have been searched) 67150 19. #19 ("Resistance Training"):ti,ab,kw OR ("Muscle Stretching Exercises"):ti,ab,kw OR ("Strength Training"):ti,ab,kw OR ("Tai Chi"):ti,ab,kw OR ("jogging"):ti,ab,kw (Word variations have been searched) 19440 20. #20 (yoga):ti,ab,kw OR (riding):ti,ab,kw OR (swimming):ti,ab,kw OR (training):ti,ab,kw OR (baduanjin):ti,ab,kw (Word variations have been searched) 157120 21. #21 (sports):ti,ab,kw OR ("Eight-section Brocade"):ti,ab,kw (Word variations have been searched) 13671 22. #22 #14 OR #15 OR #16 OR #17 OR #18 OR #19 OR #20 OR #21 220789 23. #23 #6 AND #13 AND #22 3263 |

# Supplementary file 2: Characteristics of included studies

| Study ID | Age  (Mean±SD) | Sample Size  (M/F) | Muscle mass | BMI | Diagnosis of  Sarcopenia | Intervention detail |
| --- | --- | --- | --- | --- | --- | --- |
| Ana C. da Silva, et al. (2016) | RT: 63.0±6.6  CON:69.5±5.7 | RT: 39 (12/27)  CON: 32 (6/26) | SMI  RT: 8.7±1.6  CON: 8.1±1.8 | RT: 28.0±4.8  CON: 27.8±5.4 | ECS  HG<27 kg for men, <16 kg for women; MMI<10.76 kg/m^2^ for men, < 6.76 kg/m^2^ for women.  MMI: RT: 8.7±1.6; CON: 8.1±1.8 | RT: 1h, based on machine or barbell, 3 times/week, 11 exercises, 6-15 reps/set, 60%-85% 1RM. |
| Yaqin Xiao. (2023) | RT: 53.9±14.5  CON: 51.2±12.9 | RT: 30 (13/17)  CON: 30 (17/13) | SMM:  RT: 22.7±7.0  CON: 24.1±8.6 | RT: 17.2±2.2  CON:17.0±2.3 | AWGS  ①ASMI<7.0 kg/m^2^ in men,<5.7 kg/m^2^ in women; HG<28 kg in men and <18 kg in women; or speed＜1.0 m/s.  HG: RT:18.5±4.9; CON:19.1±5.9 ASMI: RT:5.42±0.73; CON:5.64±0.81 | RT: based on resistance band, 6 times/week, 4 exercises, 30 reps/set, 3 set/day, 6-9 OMNI. |
| Hung-Ting Chen et al. (2018) | RT: 66.7±5.3  CON: 68.3±2.8 | RT: 17 (0/17)  CON:16 (0/16) | SMM  RT: 20.99±3.00  CON:19.99±2.74 | NA | AWGS  ASMI<5.7 kg/m^2^, HG<18 kg. ASMI:RT:5.57±0.28; CON:5.45±0.29  HG: RT:17.51±3.80; CON:17.93±4.82 | RT: 60 min, based on kettlebell, 2 times/week, 11 exercises, 8-12 reps/set, 3 sets, 60%-70% 1RM, 2-3 rests. |
| Yu-Hao Lee et al. (2021) | RT: 70.13±4.41  CON: 71.82±5.23 | RT: 15 (0/15)  CON: 12 (0/12) | SMI  RT: 5.01±0.83  CON: 5.12±1 | RT: 26.95±3.31  CON:28.93±3.55 | AMI＜5.67 kg/m^2^ and HG <20 kg or speed <0.8 m/s.  GS: RT:0.82±0.21; CON:0.90±0.21  HG: RT:20.40±4.00; CON:19.34±6.36 | RT: 40 min, based on elastic band, 3 times/week, 5 parts major muscle, 10 reps/set, 3 sets, 13 RPE. |
| Sanna Vikberg et al. (2018) | RT: 70.9±0.28  CON: 70.0±0.29 | RT: 36 (16/20)  CON: 34 (16/18) | AMI  RT: 6.25±0.86  CON: 6.24±0.85 | RT: 22.72±2.35  CON: 23.33±3.01 | EWGSOP  AMI≤7.29 (range:5.69-7.29) among men, ≤5.93(range:4.50-5.93) among women. | RT: 45 min, based on one's body weight, 8 exercises, 3 times/week, 10-12 reps/set, 2-4 sets, CR-10 6-7. |
| Zhi‑Juan Dong et al. (2019) | RT: 59.0(32.5,66.5)  CON: 62.5(50.5,70.0) | RT: 21 (9/12)  CON: 20 (12/8) | SMM  RT: 21.19 ±3.65  CON: 1.06±3.12 | RT: 18.96±3.08  CON: 20.49±3.41 | AWGS  SMI:male < 7.0 kg/m^2^, female < 5.7 kg/m^2^; HG:(male < 26 kg, female < 18 kg), speed (< 0.8 m/s)  SMI: RT:5.70±0.80; CON:5.87 ±0.69 | RT: 1-2h, based on own body weight and elastic balls, 3 times/week, 10 reps/set, 10sets, 0.5 kg/week, 0-5 kg weight. |
| Minoru Yamada et al. (2019) | RT: 84.7±5.1  CON: 83.9±5.7 | RT: 28 (10/18)  CON: 28 (13/15) | AMM  RT: 17.19±4.58  CON: 17.63±5.11 | RT: 22.6±3.0  CON: 21.2±2.9 | AWGS | RT: 20 min, based on body weight or elastic band, 2times/week, 7 exercises, 20 reps/set, 3 sets. |
| André Bonadias Gadelha et al. (2021) | RT: 65.0±3.6  CON: 63.8±4.1 | RT: 37 (-/-)  CON: 28 (-/-) | NA | RT: 20.8±5.7  CON: 20.7±4.7 | EWGSOP-2  FFMI ＜15.3 and 15.6 kg/m^2^ for men and women; handgrip strength＜30 kg and 20 kg for men and women  HG: RT:23.1±2.2; CON:25.1±3.0 | RT: 40 min, based on one's body weight, 6 exercises, 8-12 reps/set, 3 sets, 5-8 OMNI, 2 min rest. |
| Myong-Won Seo et al. (2021) | RT: 70.3±5.38  CON: 72.9±4.75 | RT: 12 (0/12)  CON: 10 (0/10) | AMM  RT: 12.3±0.96  CON: 12.4±0.95 | RT: 22.9±2.02  CON: 22.4±1.52 | EWGSOP  (1) GS< 1.0 m·s^−1^ and ASMI < 5.67 kg·m^−2^, or GS > 1.0 m·s^−1^, (2) grip strength<20 kg, ASMI<5.67 kg·m^−2^.  (3) PBF< 35%; (4) T-score < −2.5 | RT: 50 min, based on elastic band, 17 exercises, 3 times/week, 6-15 reps, 3-5 sets, 4-8 OMNI Scale, 1 min rest. |
| Yu-Hsuan Chien et al. (2022) | RT: 67.6±7.7  CON: 67.3±6.1 | RT: 20 (5/15)  CON: 20 (2/18) | AMI  RT: 6.9±0.9  CON: 6.9±0.7 | RT:2 4.3±3.4  CON: 25.5±3.7 | AWGS: CC<34 cm, GS<28 kg for males.CC<33 cm and GS <18 kg for females.  CC: RT:30.4±2.3; CON:31.3±2.0 GS: RT:15.9±5.0; CON:15.1±3.8 | RT: 30min, based on Sandbags, 5 exercises, 3 times/week, 8-15 reps, 3 sets, 1-2min rest, 13 RPE. |
| Helton de Sá Souza et al. (2022) | RT: 77.42±6.25  CON: 74.64±7.13 | RT: 14(7/7)  CON: 14(3/11) | AMI (man)  RT: 7.51±0.48 CON: 7.13±0.44  AMI (woman):  RT: 5.85±0.57  CON: 6.28±0.73 | RT: 25.54±2.04  CON: 26.78±4.44 | AMI<7.27 g/m^2^、HG <40 kg、SPPB-score <6 points in man, AMI<5 g/m^2^, HG<30 kg, SPPB score<6 points in women.  SPPB(men) RT: 10.0±1.15; CON: 10.0±1.73  SPPB(women): RT: 5.85±0.57; CON: 8.63±2.73  HG(men)  RT: 26.41±5.10; CON: 27.66±4.93  HG(women)  RT: 16.57±2.50; CON: 18.82±6.65 | RT: based on one's own weight, 3 times/week, 8 exercises, 2 sets, 10-12 reps, 50%-75% 1RM. |
| Hung-Ting Chen et al. (2017) | RT: 68.9±4.4  CON: 68.6±3.1 | RT: 15(3/12)  CON: 15(2/13) | SMM  RT: 22.9±4.0  CON: 21.6±3.6 | RT: 28.3±4.4  CON: 29.0±3.9 | ASM(kg)/Weight (kg)*100%:  men ≤32.5%; women≤25.7%.  RT: 24.1±2.4; CON:23.0±2.6 | RT: 2 times/week, 10 exercises, 60-70% 1RM, 8–12 reps/set, 3 sets, 2–3 min rest, from seated to standing exercises. |
| Chun-De Liao et al. (2017) | RT: 66.39±4.49  CON: 68.42±5.86 | RT: 25(0/25)  CON: 21(0/21) | SMI  RT: 6.85±0.33  CON: 6.91±0.24 | RT: 27.32±3.33  CON: 28.19±3.27 | EWGS  SMI≤7.15kg/m^2^.  RT: 6.85±0.33; CON: 6.91±0.24 | RT: 35-40 min, based on the band, 6 exercises, 10-20 reps/set, 3 sets, 10-13 RPE. |

*Note.* M/F: Male or Female; RT: Resistance training; CON: control group; SMI: Skeletal muscle mass index= SMM/height2; SMM: Skeletal muscle mass; ASM: Appendicular skeletal muscle mass, ASI=ASM/height^2^=ASMI; ECS: European Consensus on Sarcopenia; EWGSOP: European Working Group on Sarcopenia in Older People=EWGS; AWGS: Asian Working Group for Sarcopenia; CC: calf circumference; SPPB: Short physical performance battery; GS: gait speed. PBF: percentage body fat; BMI: Body Mass Index; NA: Not available.

# Supplementary file 3: Key assumptions of Network Meta-Analysis

First, we plotted the observed effects of different resistance training doses on muscle strength. Based on the observed shapes, a range of recommended non-linear functions (i.e., Emax, restricted cubic splines, quadratic, and non-monotonically up^[1]^ were used to model the data. Next, we derived and compared different fit indices^[2]^ (i.e., Deviance Information Criterion (DIC), between-study standard deviation, number of parameters in the model, residual values) as well as corresponding deviance plots^[2]^ across models (Supplementary File 4). Restricted cubic splines yielded the best fit in all cases and were therefore used to assess the non-linear dose-response associations (Supplementary File 4). According to the model with the best fit (Supplementary Table 3) and biological plausibility^[1]^, we placed three knots at the 10th, 50th and 90th percentile of the exercise dose^[3, 4]^. Departure from linearity was assessed using a Wald test^[3, 4]^. Beta coefficients from the restricted cubic splines were used to estimate the resistance training dose at which the predicted maximal significant effect on handgrip strength was achieved. This information was used to rank the doses of resistance training based on their probability to elicit changes on muscle strength, from worst to best.


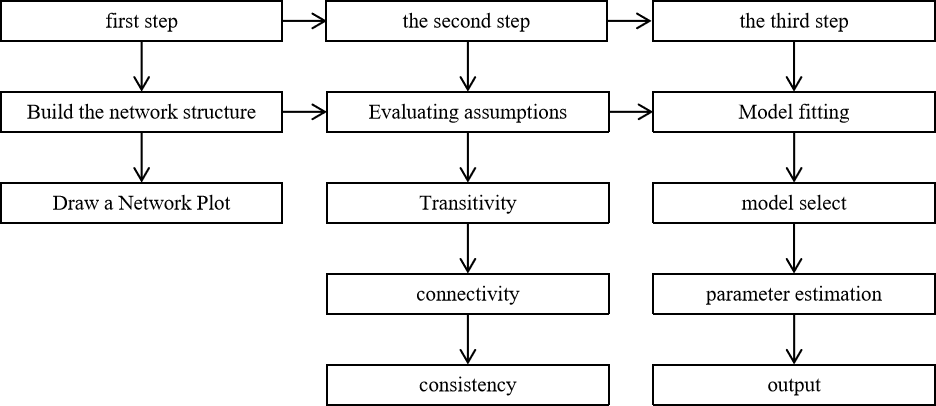


### **Supplementary Figure 1.** Schematic diagram of network meta-statistics analysis

There are three key assumptions to conduct a Network Meta-Analysis (NMA): (1) network connectivity, (2) consistency in the data, and (3) transitivity.

*Connectivity*

Connectivity is a key assumption in NMA which if deemed insufficient (i.e., due to lack of direct comparators) can lead to low statistical power and misleading results (ter Veer E, et al., 2019). In our study, we assessed connectivity of the network at both treatment and agent levels visually and found no evidence of unconnectedness on either network (Supplementary Figure 1).

### **Supplementary Figure 2.** Treatment-level network.


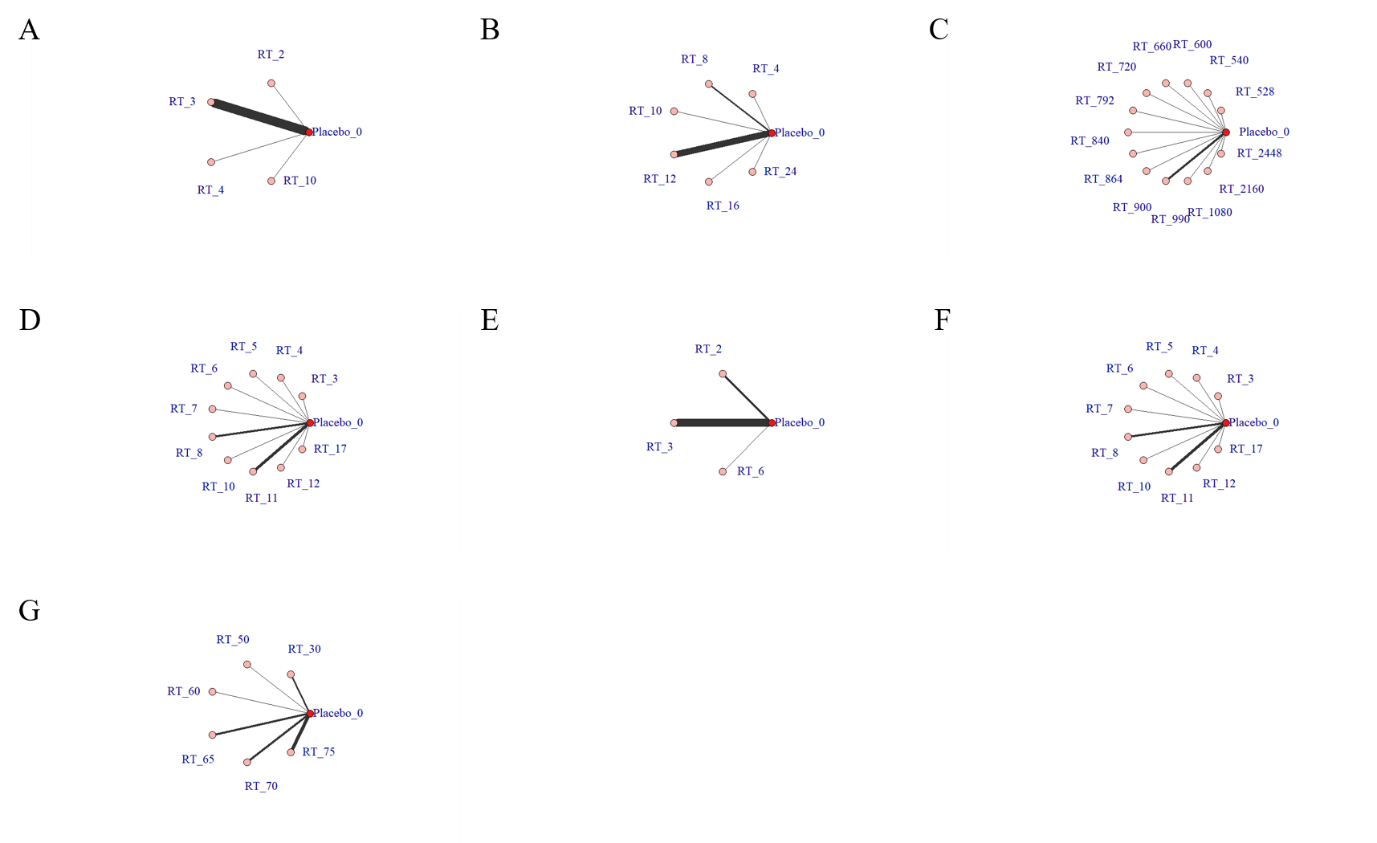


**Supplementary Figure 1**. Network plot of study limitations of the included studies. (A): Sets; (B): Period; (C): Reps/week; (D): Reps; (E): Frequency; (F): Exercises; (G): Intensity

*Consistency*

We carried out consistency analysis in the data through the comparison of consistent (i.e., network effect sizes) and unrelated mean effects (UME) models (i.e., pairwise effect sizes) of the network. In practice, we checked whether deviance, the number of estimated parameters in the network, and the Deviance Informative Criterion (DIC) indicators were similar for both models which would indicate a good fit (Wheeler DC, et al., 2010). Comparison of these parameters indicated good consistency across models (Supplementary Table 1).

### **Supplementary Table 1.** Consistent and UME models fit comparison

**Table 1.1.** Consistent and UME models fit comparison (Sets)

| **Model** | **pD** | **Residual deviance** | **Deviance** | **DIC** | **SD** |
| --- | --- | --- | --- | --- | --- |
| Consistent | 28.0 | 29.565 | 98.783 | 126.1 | 3.984 |
| UME | 27.9 | 28.705 | 97.923 | 125.1 | 5.329 |

**Table 1.2.** Consistent and UME models fit comparison (Period)

| **Model** | **pD** | **Residual deviance** | **Deviance** | **DIC** | **SD** |
| --- | --- | --- | --- | --- | --- |
| Consistent | 28.2 | 29.290 | 98.508 | 125.9 | 4.338 |
| UME | 27.2 | 28.240 | 97.459 | 124.0 | 3.836 |

**Table 1.3.** Consistent and UME models fit comparison (Reps/week)

| **Model** | **pD** | **Residual deviance** | **Deviance** | **DIC** | **SD** |
| --- | --- | --- | --- | --- | --- |
| Consistent | 29.4 | 30.755 | 99.973 | 128.6 | 6.689 |
| UME | 28.0 | 28.863 | 98.081 | 125.3 | 5.114 |

**Table 1.4.** Consistent and UME models fit comparison (Reps)

| **Model** | **pD** | **Residual deviance** | **Deviance** | **DIC** | **SD** |
| --- | --- | --- | --- | --- | --- |
| Consistent | 27.3 | 29.181 | 98.399 | 125.2 | 4.614 |
| UME | 27.4 | 28.853 | 98.071 | 124.7 | 3.958 |

**Table 1.5** Consistent and UME models fit comparison (Frequency)

| **Model** | **pD** | **Residual deviance** | **Deviance** | **DIC** | **SD** |
| --- | --- | --- | --- | --- | --- |
| Consistent | 27.3 | 29.181 | 98.399 | 125.2 | 3.984 |
| UME | 27.4 | 28.853 | 98.071 | 124.7 | 3.958 |

**Table 1.6.** Consistent and UME models fit comparison (Exercises)

| **Model** | **pD** | **Residual deviance** | **Deviance** | **DIC** | **SD** |
| --- | --- | --- | --- | --- | --- |
| Consistent | 29.6 | 29.625 | 98.843 | 127.8 | 6.318 |
| UME | 26.5 | 28.800 | 98.018 | 123.9 | 3.590 |

**Table 1.7** Consistent and UME models fit comparison (Intensity)

| **Model** | **pD** | **Residual deviance** | **Deviance** | **DIC** | **SD** |
| --- | --- | --- | --- | --- | --- |
| Consistent | 29.0 | 30.005 | 99.223 | 127.5 | 4.717 |
| UME | 27.5 | 28.891 | 98.109 | 125.1 | 4.291 |

*Note.* pD: Number of estimated parameters; DIC: Deviance Informative Criterion; SD: Standard Deviation; UME: Unrelated Mean Effects. Scientific literature indicated that the main indicator to assess the model fit is the DIC. As lower DIC, better fit.

*Transitivity*

We assessed transitivity via MBNMA node-splitting approach. This method splits and compares contributions for a particular treatment contrast into direct and indirect evidence (van Valkenhoef et al., 2016). Similar effects denote good transitivity. Supplementary Figures 2 and Table 2 below present the results for transitivity in this meta-analysis.

### **Supplementary Table 2**. Node-splitting analysis of inconsistency

Table 2.1. Node-splitting analysis of inconsistency (Sets)

| **Comparison** | **p-value** | **Median** | **2.50%** | **97.50%** |
| --- | --- | --- | --- | --- |
| RT_10 vs Placebo_0 | 0.189 |  |  |  |
| -> direct |  | 4.723 | -5.144 | 13.749 |
| -> indirect |  | 16.14 | 7.624 | 23.365 |
| -> MBNMA |  | 10.957 | 4.474 | 17.637 |
|  |  |  |  |  |
| RT_4 vs Placebo_0 | 0.496 |  |  |  |
| -> direct |  | 6.831 | -4.114 | 18.086 |
| -> indirect |  | 5.084 | 1.389 | 8.282 |
| -> MBNMA |  | 5.283 | 2.047 | 8.552 |
|  |  |  |  |  |
| RT_3 vs Placebo_0 | 0.218 |  |  |  |
| -> direct |  | 5.935 | 2.604 | 8.832 |
| -> indirect |  | 2.108 | -0.505 | 5.595 |
| -> MBNMA |  | 4.091 | 1.557 | 7.068 |
|  |  |  |  |  |
| RT_2 vs Placebo_0 | 0.177 |  |  |  |
| -> direct |  | 9.688 | -1.265 | 21.134 |
| -> indirect |  | 2.637 | 0.756 | 4.926 |
| -> MBNMA |  | 2.809 | 1.053 | 5.582 |

Table 2.2. Node-splitting analysis of inconsistency (Period)

| **Comparison** | **p-value** | **Median** | **2.50%** | **97.50%** |
| --- | --- | --- | --- | --- |
| RT_24 vs Placebo_0 | 0.446 |  |  |  |
| -> direct |  | 7.102 | -0.662 | 14.943 |
| -> indirect |  | 11.617 | 6.715 | 15.706 |
| -> MBNMA |  | 10.307 | 6.4 | 14.217 |
|  |  |  |  |  |
| RT_16 vs Placebo_0 | 0.556 |  |  |  |
| -> direct |  | 7.128 | -1.261 | 15.761 |
| -> indirect |  | 7.725 | 4.561 | 10.611 |
| -> MBNMA |  | 7.614 | 4.924 | 10.226 |
|  |  |  |  |  |
| RT_12 vs Placebo_0 | 0.259 |  |  |  |
| -> direct |  | 7.567 | 4.638 | 10.282 |
| -> indirect |  | 4.37 | 1.465 | 7.328 |
| -> MBNMA |  | 6.043 | 3.871 | 8.097 |
|  |  |  |  |  |
| RT_10 vs Placebo_0 | 0.309 |  |  |  |
| -> direct |  | 1.623 | -7.348 | 10.637 |
| -> indirect |  | 5.382 | 3.265 | 7.399 |
| -> MBNMA |  | 5.189 | 3.302 | 7.118 |
|  |  |  |  |  |
| RT_8 vs Placebo_0 | 0.484 |  |  |  |
| -> direct |  | 4.11 | -2.306 | 10.466 |
| -> indirect |  | 4.291 | 2.59 | 6.262 |
| -> MBNMA |  | 4.279 | 2.703 | 6.029 |
|  |  |  |  |  |
| RT_4 vs Placebo_0 | 0.262 |  |  |  |
| -> direct |  | 2.089 | -6.78 | 10.875 |
| -> indirect |  | 2.247 | 1.337 | 3.736 |
| -> MBNMA |  | 2.253 | 1.402 | 3.76 |

Table 2.3. Node-splitting analysis of inconsistency (Frequency)

| **Comparison** |  | **p-value** | **Median** | **2.50%** | **97.50%** |
| --- | --- | --- | --- | --- | --- |
| RT_6 vs Placebo_0 |  | 0.146 |  |  |  |
| -> direct |  |  | 2.22 | -6.108 | 10.971 |
| -> indirect |  |  | 12.181 | 6.686 | 16.626 |
| -> MBNMA |  |  | 9.207 | 3.889 | 13.935 |
|  |  |  |  |  |  |
| RT_3 vs Placebo_0 |  | 0.189 |  |  |  |
| -> direct |  |  | 6.723 | 3.444 | 9.756 |
| -> indirect |  |  | 2.033 | -1.563 | 6.188 |
| -> MBNMA |  |  | 5.011 | 2.011 | 7.712 |
|  |  |  |  |  |  |
| RT_2 vs Placebo_0 |  | 0.415 |  |  |  |
| -> direct |  |  | 5.57 | -0.193 | 11.724 |
| -> indirect |  |  | 3.251 | 1.104 | 5.394 |
| -> MBNMA |  |  | 3.435 | 1.355 | 5.572 |

Table 2.4. Node-splitting analysis of inconsistency (Reps/week)

| **Comparison** | **p-value** | **Median** | **2.50%** | **97.50%** |
| --- | --- | --- | --- | --- |
| RT_2448 vs Placebo_0 | 0.513 |  |  |  |
| -> direct |  | 7.057 | -1.905 | 15.512 |
| -> indirect |  | 6.492 | 3.736 | 9.143 |
| -> MBNMA |  | 6.582 | 3.888 | 9.119 |
|  |  |  |  |  |
| RT_2160 vs Placebo_0 | 0.272 |  |  |  |
| -> direct |  | 2.21 | -5.956 | 10.51 |
| -> indirect |  | 6.996 | 4.332 | 9.455 |
| -> MBNMA |  | 6.553 | 3.877 | 9.064 |
|  |  |  |  |  |
| RT_1080 vs Placebo_0 | 0.473 |  |  |  |
| -> direct |  | 7.195 | -1.546 | 15.663 |
| -> indirect |  | 6.204 | 3.523 | 8.645 |
| -> MBNMA |  | 6.341 | 3.745 | 8.703 |
|  |  |  |  |  |
| RT_990 vs Placebo_0 | 0.033 |  |  |  |
| -> direct |  | 11.485 | 7.464 | 14.381 |
| -> indirect |  | 5.124 | 3.324 | 6.831 |
| -> MBNMA |  | 6.304 | 3.722 | 8.642 |
|  |  |  |  |  |
| RT_900 vs Placebo_0 | 0.437 |  |  |  |
| -> direct |  | 4.591 | -4.299 | 13.318 |
| -> indirect |  | 6.308 | 3.81 | 8.684 |
| -> MBNMA |  | 6.25 | 3.702 | 8.547 |
|  |  |  |  |  |
| RT_864 vs Placebo_0 | 0.406 |  |  |  |
| -> direct |  | 4.01 | -5.175 | 12.995 |
| -> indirect |  | 6.362 | 3.843 | 8.81 |
| -> MBNMA |  | 6.234 | 3.689 | 8.516 |
|  |  |  |  |  |
| RT_840 vs Placebo_0 | 0.437 |  |  |  |
| -> direct |  | 8.225 | -0.024 | 17.463 |
| -> indirect |  | 6.031 | 3.409 | 8.48 |
| -> MBNMA |  | 6.217 | 3.674 | 8.498 |
|  |  |  |  |  |
| RT_792 vs Placebo_0 | 0.279 |  |  |  |
| -> direct |  | 1.555 | -8.467 | 10.973 |
| -> indirect |  | 6.443 | 4.018 | 8.847 |
| -> MBNMA |  | 6.19 | 3.673 | 8.474 |
|  |  |  |  |  |
| RT_720 vs Placebo_0 | 0.25 |  |  |  |
| -> direct |  | 2.2 | -13.824 | 17.346 |
| -> indirect |  | 6.177 | 3.905 | 8.408 |
| -> MBNMA |  | 6.133 | 3.649 | 8.395 |
|  |  |  |  |  |
| RT_660 vs Placebo_0 | 0.432 |  |  |  |
| -> direct |  | 4.227 | -4.954 | 12.626 |
| -> indirect |  | 6.156 | 3.616 | 8.473 |
| -> MBNMA |  | 6.083 | 3.624 | 8.319 |
|  |  |  |  |  |
| RT_600 vs Placebo_0 | 0.364 |  |  |  |
| -> direct |  | 3.706 | -6.107 | 14.408 |
| -> indirect |  | 6.069 | 3.677 | 8.311 |
| -> MBNMA |  | 6.025 | 3.58 | 8.254 |
|  |  |  |  |  |
| RT_540 vs Placebo_0 | 0.346 |  |  |  |
| -> direct |  | 2.547 | -6.045 | 11 |
| -> indirect |  | 6.109 | 3.776 | 8.331 |
| -> MBNMA |  | 5.945 | 3.511 | 8.179 |
|  |  |  |  |  |
| RT_528 vs Placebo_0 | 0.298 |  |  |  |
| -> direct |  | 9.97 | 0.714 | 19.614 |
| -> indirect |  | 5.61 | 3.259 | 7.927 |
| -> MBNMA |  | 5.923 | 3.496 | 8.161 |

Table 2.5. Node-splitting analysis of inconsistency (Intensity)

| **Comparison** |  | **p-value** | **Median** | **2.50%** | **97.50%** |
| --- | --- | --- | --- | --- | --- |
| RT_75 vs Placebo_0 |  | 0.749 |  |  |  |
| -> direct |  |  | 7.298 | 2.879 | 11.526 |
| -> indirect |  |  | 6.094 | 2.522 | 9.806 |
| -> MBNMA |  |  | 6.588 | 3.754 | 9.198 |
|  |  |  |  |  |  |
| RT_70 vs Placebo_0 |  | 0.499 |  |  |  |
| -> direct |  |  | 4.501 | -0.631 | 9.936 |
| -> indirect |  |  | 7.017 | 3.888 | 10.04 |
| -> MBNMA |  |  | 6.389 | 3.651 | 8.874 |
|  |  |  |  |  |  |
| RT_65 vs Placebo_0 |  | 0.677 |  |  |  |
| -> direct |  |  | 5.92 | -0.037 | 11.411 |
| -> indirect |  |  | 6.197 | 3.224 | 8.899 |
| -> MBNMA |  |  | 6.18 | 3.496 | 8.558 |
|  |  |  |  |  |  |
| RT_60 vs Placebo_0 |  | 0.265 |  |  |  |
| -> direct |  |  | 2.578 | -13.879 | 18.208 |
| -> indirect |  |  | 6.004 | 3.554 | 8.474 |
| -> MBNMA |  |  | 5.937 | 3.387 | 8.256 |
|  |  |  |  |  |  |
| RT_50 vs Placebo_0 |  | 0.432 |  |  |  |
| -> direct |  |  | 4.633 | -4.985 | 14.256 |
| -> indirect |  |  | 5.324 | 2.961 | 7.762 |
| -> MBNMA |  |  | 5.363 | 3.013 | 7.664 |
|  |  |  |  |  |  |
| RT_30 vs Placebo_0 |  | 0.244 |  |  |  |
| -> direct |  |  | 7.713 | 1.59 | 13.84 |
| -> indirect |  |  | 3.461 | 1.884 | 5.861 |
| -> MBNMA |  |  | 3.877 | 2.118 | 6.585 |

Table 2.6. Node-splitting nalysis of inconsistency (Reps)

| **Comparison** |  | **p-value** | **Median** | **2.50%** | **97.50%** |
| --- | --- | --- | --- | --- | --- |
| RT_30 vs Placebo_0 |  | 0.145 |  |  |  |
| -> direct |  |  | 2.15 | -7.495 | 10.883 |
| -> indirect |  |  | 12.664 | 6.783 | 18.447 |
| -> MBNMA |  |  | 9.431 | 4.533 | 14.843 |
|  |  |  |  |  |  |
| RT_20 vs Placebo_0 |  | 0.571 |  |  |  |
| -> direct |  |  | 8.351 | -2.664 | 18.895 |
| -> indirect |  |  | 7.089 | 2.98 | 11.329 |
| -> MBNMA |  |  | 7.354 | 3.54 | 10.937 |
|  |  |  |  |  |  |
| RT_16 vs Placebo_0 |  | 0.462 |  |  |  |
| -> direct |  |  | 3.877 | -6.895 | 15.063 |
| -> indirect |  |  | 6.456 | 2.926 | 9.907 |
| -> MBNMA |  |  | 6.362 | 2.994 | 9.323 |
|  |  |  |  |  |  |
| RT_12 vs Placebo_0 |  | 0.606 |  |  |  |
| -> direct |  |  | 4.821 | -2.23 | 12.446 |
| -> indirect |  |  | 5.127 | 2.106 | 8.349 |
| -> MBNMA |  |  | 5.19 | 2.354 | 7.884 |
|  |  |  |  |  |  |
| RT_11 vs Placebo_0 |  | 0.537 |  |  |  |
| -> direct |  |  | 5.604 | -2.326 | 13.668 |
| -> indirect |  |  | 4.607 | 1.853 | 7.558 |
| -> MBNMA |  |  | 4.857 | 2.169 | 7.536 |
|  |  |  |  |  |  |
| RT_10 vs Placebo_0 |  | 0.149 |  |  |  |
| -> direct |  |  | 7.292 | 3.817 | 10.615 |
| -> indirect |  |  | 2.958 | 0.589 | 5.554 |
| -> MBNMA |  |  | 4.497 | 1.995 | 7.254 |

Table 2.7. Node-splitting analysis of inconsistency (Exercises)

| Comparison | **p-value** | **Median** | **2.50%** | **97.50%** |
| --- | --- | --- | --- | --- |
| RT_17 vs Placebo_0 | 0.394 |  |  |  |
| -> direct |  | 6.983 | -0.147 | 14.05 |
| -> indirect |  | 11.535 | 7.199 | 15.174 |
| -> MBNMA |  | 10.368 | 6.628 | 13.751 |
|  |  |  |  |  |
| RT_12 vs Placebo_0 | 0.569 |  |  |  |
| -> direct |  | 7.178 | -1.042 | 15.06 |
| -> indirect |  | 7.845 | 4.59 | 10.639 |
| -> MBNMA |  | 7.821 | 5.053 | 10.295 |
|  |  |  |  |  |
| RT_11 vs Placebo_0 | 0.36 |  |  |  |
| -> direct |  | 9.151 | 5.034 | 12.751 |
| -> indirect |  | 6.164 | 3.042 | 8.948 |
| -> MBNMA |  | 7.273 | 4.692 | 9.561 |
|  |  |  |  |  |
| RT_10 vs Placebo_0 | 0.353 |  |  |  |
| -> direct |  | 3.629 | -5.481 | 12.395 |
| -> indirect |  | 6.866 | 4.28 | 9.07 |
| -> MBNMA |  | 6.709 | 4.326 | 8.852 |
|  |  |  |  |  |
| RT_8 vs Placebo_0 | 0.527 |  |  |  |
| -> direct |  | 5.194 | -1.157 | 10.973 |
| -> indirect |  | 5.574 | 3.469 | 7.602 |
| -> MBNMA |  | 5.535 | 3.552 | 7.388 |
|  |  |  |  |  |
| RT_7 vs Placebo_0 | 0.277 |  |  |  |
| -> direct |  | 8.354 | 0.739 | 15.769 |
| -> indirect |  | 4.738 | 2.874 | 6.432 |
| -> MBNMA |  | 4.907 | 3.142 | 6.636 |
|  |  |  |  |  |
| RT_6 vs Placebo_0 | 0.372 |  |  |  |
| -> direct |  | 3.887 | -4.42 | 11.958 |
| -> indirect |  | 4.291 | 2.701 | 6.101 |
| -> MBNMA |  | 4.26 | 2.72 | 5.844 |
|  |  |  |  |  |
| RT_5 vs Placebo_0 | 0.333 |  |  |  |
| -> direct |  | 2.591 | -5.269 | 10.307 |
| -> indirect |  | 3.589 | 2.182 | 5.224 |
| -> MBNMA |  | 3.596 | 2.291 | 5.068 |
|  |  |  |  |  |
| RT_4 vs Placebo_0 | 0.29 |  |  |  |
| -> direct |  | 2.191 | -6.228 | 10.167 |
| -> indirect |  | 2.911 | 1.867 | 4.323 |
| -> MBNMA |  | 2.91 | 1.851 | 4.279 |
|  |  |  |  |  |
| RT_3 vs Placebo_0 | 0.201 |  |  |  |
| -> direct |  | 4.762 | -3.475 | 13.084 |
| -> indirect |  | 2.178 | 1.293 | 3.241 |
| -> MBNMA |  | 2.211 | 1.406 | 3.379 |

### **Supplementary Figure 3.** Node-splitting analysis (density plot).

**
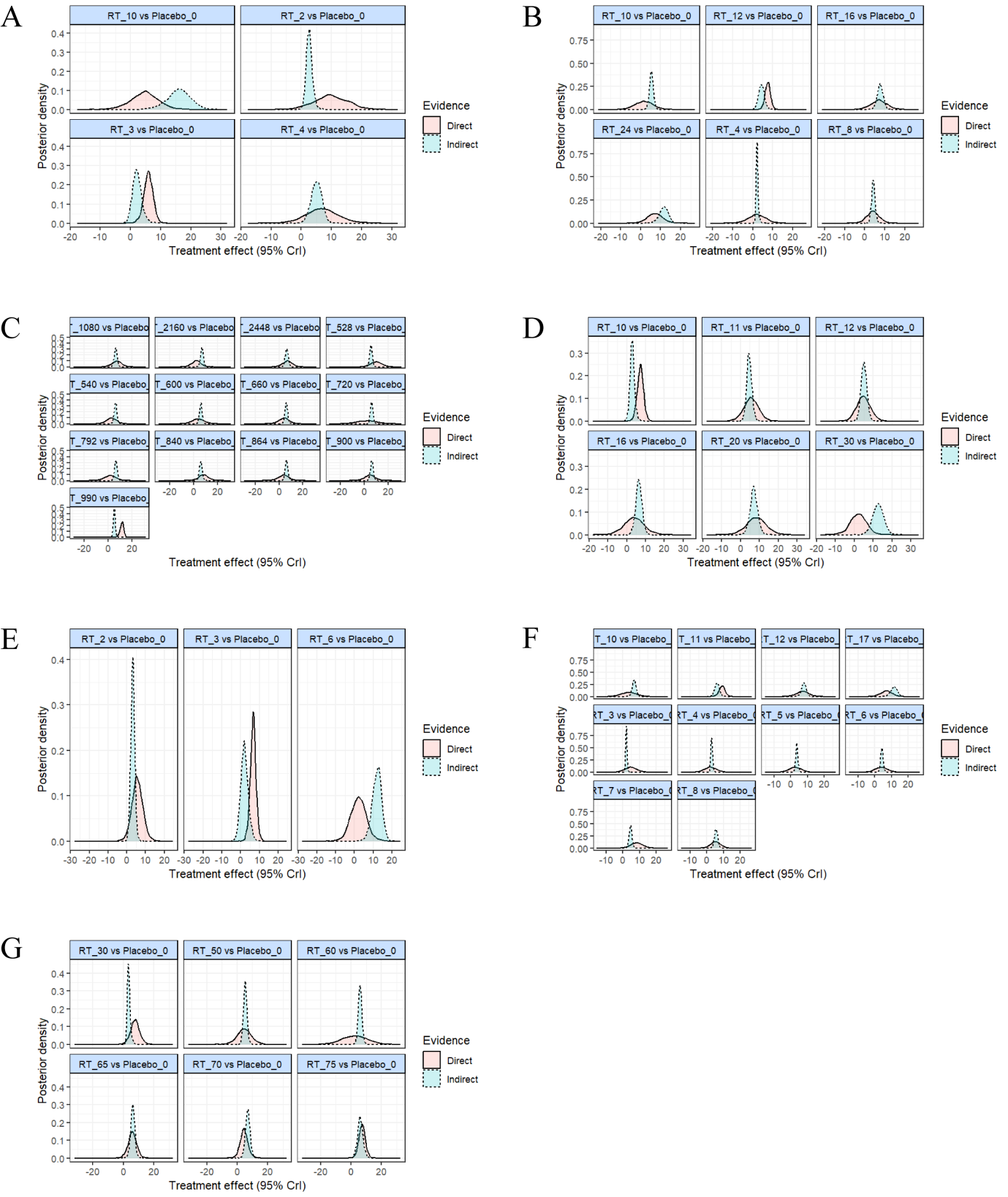
**

**Supplementary Figures 3.** Node-splitting analysis (density plot). (A): Sets; (B): Period; (C): Reps/week; (D): Reps; (E): Frequency; (F): Exercises; (G): Intensity

# Supplementary file 4: Non-linear functions and models fit comparison

The different doses of physical activity were meta-analysed as independent and unrelated treatments (i.e., “split” NMA). This step is useful to determine which function fits the data better and should subsequently be used in a Model-Based Network Meta-Analysis (MBNMA) (Pedder, 2021). Supplementary Figure 3 shows the different responses (Hedges’ g) of each dose for overall, respectively.

### **Supplementary Figure 4.** “Split” NMA of different Exercise dose.

**
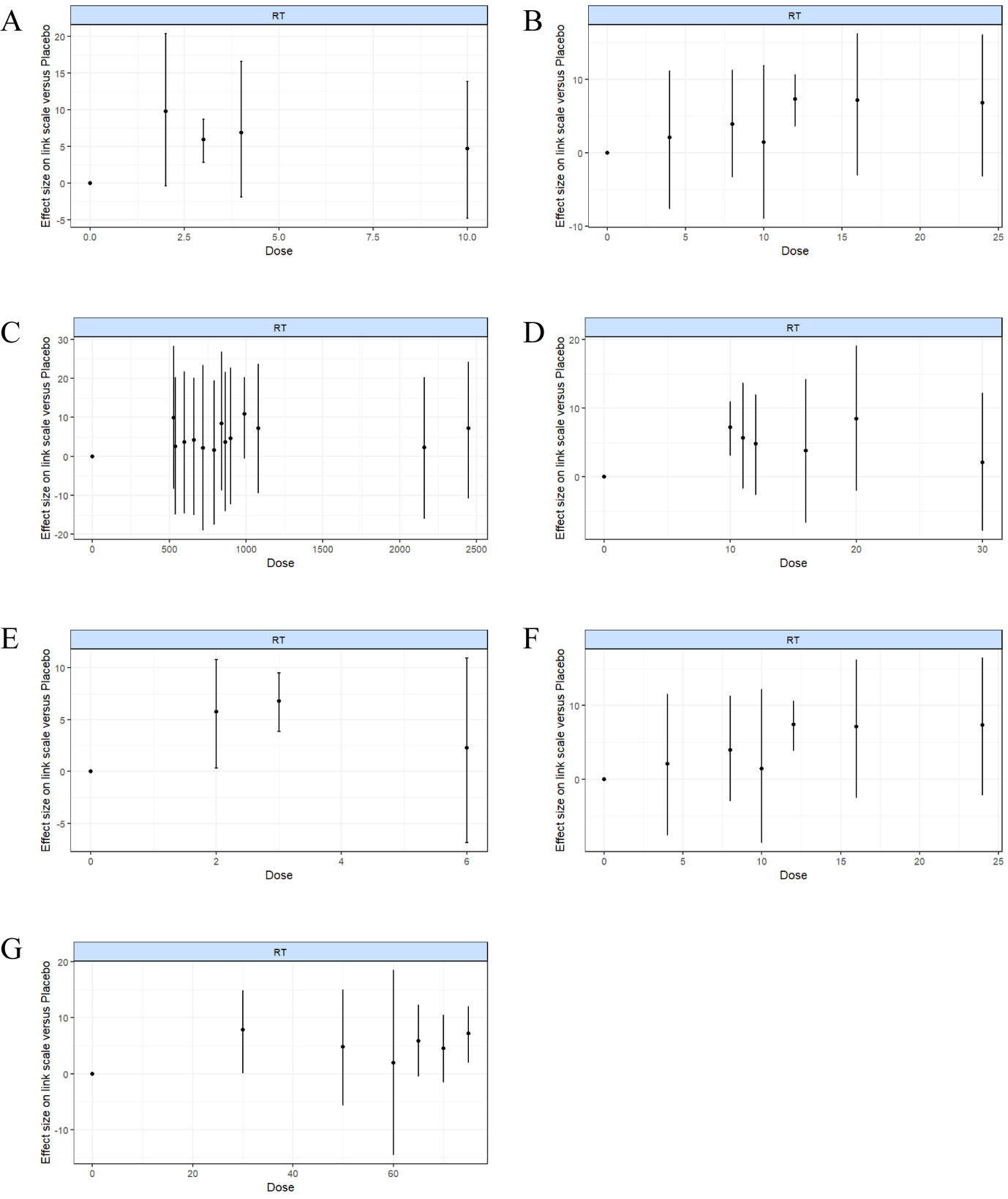
**

**Supplementary Figure 4.** “Split” NMA of different Exercise dose. Non-linear functions and models fit comparison. (A): Sets; (B): Period; (C): Reps/week; (D): Reps; (E): Times; (F): Exercises; (G): Intensity

Supplementary Table 3 shows the fit indices from each of the models fitted. For our data, restricted cubic splines show the best fit and were therefore used in subsequent analyses.

### **Supplementary Table 3.** Models fit comparison

| Table 3 shows the fit indices from each of the models fitted. For our data, restricted cubic splines show the best fit and were therefore used in subsequent analyses.  **Table 3.1.** Models fit comparison (Sets)   \| **Model** \| **DIC** \| **SD** \| **Deviance** \| **Residual deviance** \| **pD** \| \| --- \| --- \| --- \| --- \| --- \| --- \| \| **Emax**  **(common treatment effects)** \| 209.4 \| NA \| 193.921 \| 124.702 \| 16.1 \| \| **Emax**  **(RANDOM treatment effects)** \| 125.1 \| 5.016 \| 97.949 \| 28.730 \| 27.6 \| \| **Linear**  **(common treatment effects)** \| 319.3 \| NA \| 303.971 \| 234.753 \| 16.0 \| \| **Linear**  **(random treatment effects)** \| 125.6 \| 5.326 \| 98.294 \| 29.076 \| 28.0 \| \| **Exponential**  **(common treatment effects)** \| 206.9 \| NA \| 191.481 \| 122.263 \| 16.0 \| \| **Exponential**  **(RANDOM treatment effects)** \| 125.1 \| 4.586 \| 98.241 \| 29.022 \| 27.5 \| \| **Restricted cubic spline**  **(common treatment effects; 3 knots)** \| 210.4 \| NA \| 194.208 \| 124.989 \| 16.8 \| \| **Restricted cubic spline**  **(random treatment effects; 3 knots)** \| 124.5 \| 4.190 \| 98.049 \| 28.830 \| 27.1 \| \| **Non-parametric monotonically up**  **(common treatment effects)** \| 212.2 \| NA \| 196.485 \| 127.266 \| 16.3 \| \| **Non-parametric monotonically up**  **(RANDOM treatment effects)** \| 124.9 \| 4.580 \| 98.384 \| 29.166 \| 27.3 \|   **Table 3.2.** Models fit comparison (Period)   \| **Model** \| **DIC** \| **SD** \| **Deviance** \| **Residual deviance** \| **pD** \| \| --- \| --- \| --- \| --- \| --- \| --- \| \| **Emax**  **(common treatment effects)** \| 194.3 \| NA \| 178.764 \| 109.546 \| 16.2 \| \| **Emax**  **(RANDOM treatment effects)** \| 123.4 \| 3.719 \| 97.549 \| 28.331 \| 26.5 \| \| **Linear**  **(common treatment effects)** \| 226.5 \| NA \| 211.227 \| 142.009 \| 16.0 \| \| **Linear**  **(random treatment effects)** \| 123.8 \| 3.853 \| 97.474 \| 28.256 \| 26.9 \| \| **Exponential**  **(common treatment effects)** \| 192.1 \| NA \| 176.229 \| 107.010 \| 16.5 \| \| **Exponential**  **(RANDOM treatment effects)** \| 123.8 \| 3.848 \| 98.136 \| 28.918 \| 26.3 \| \| **Restricted cubic spline**  **(common treatment effects; 3 knots)** \| 183.5 \| NA \| 167.181 \| 97.962 \| 17.0 \| \| **Restricted cubic spline**  **(random treatment effects; 3 knots)** \| 123.9 \| 3.619 \| 97.884 \| 28.666 \| 26.6 \| \| **Non-parametric monotonically up**  **(common treatment effects)** \| 179.2 \| NA \| 162.387 \| 93.169 \| 17.5 \| \| **Non-parametric monotonically up**  **(RANDOM treatment effects)** \| 124.3 \| 3.801 \| 98.047 \| 28.829 \| 26.8 \|   **Table 3.3.** Models fit comparison (Reps/week)   \| **Model** \| **DIC** \| **SD** \| **Deviance** \| **Residual deviance** \| **pD** \| \| --- \| --- \| --- \| --- \| --- \| --- \| \| **Emax**  **(common treatment effects)** \| 206.6 \| NA \| 190.995 \| 121.777 \| 16.3 \| \| **Emax**  **(RANDOM treatment effects)** \| 124.4 \| 3.866 \| 97.932 \| 28.714 \| 27.1 \| \| **Linear**  **(common treatment effects)** \| 311.5 \| NA \| 296.226 \| 227.008 \| 16.0 \| \| **Linear**  **(random treatment effects)** \| 125.4 \| 2.027 \| 98.118 \| 28.900 \| 28.0 \| \| **Exponential**  **(common treatment effects)** \| 188.1 \| NA \| 170.020 \| 100.802 \| 18.7 \| \| **Exponential**  **(RANDOM treatment effects)** \| 123.9 \| 3.835 \| 98.095 \| 28.877 \| 26.6 \| \| **Restricted cubic spline**  **(common treatment effects; 3 knots)** \| 179.4 \| NA \| 163.265 \| 94.046 \| 16.9 \| \| **Restricted cubic spline**  **(random treatment effects; 3 knots)** \| 124.3 \| 3.723 \| 98.257 \| 29.039 \| 26.8 \| \| **Non-parametric monotonically up**  **(common treatment effects)** \| 187.0 \| NA \| 169.864 \| 100.645 \| 17.8 \| \| **Non-parametric monotonically up**  **(RANDOM treatment effects)** \| 126.2 \| 6.097 \| 98.888 \| 29.670 \| 28.0 \|   **Table 3.4.** Models fit comparison (Reps)   \| **Model** \| **DIC** \| **SD** \| **Deviance** \| **Residual deviance** \| **pD** \| \| --- \| --- \| --- \| --- \| --- \| --- \| \| **Emax**  **(common treatment effects)** \| 209.5 \| NA \| 193.787 \| 124.569 \| 16.1 \| \| **Emax**  **(RANDOM treatment effects)** \| 124.3 \| 4.773 \| 97.613 \| 28.394 \| 27.3 \| \| **Linear**  **(common treatment effects)** \| 339.2 \| NA \| 323.690 \| 254.472 \| 15.9 \| \| **Linear**  **(random treatment effects)** \| 125.1 \| 5.284 \| 97.936 \| 28.718 \| 27.7 \| \| **Exponential**  **(common treatment effects)** \| 207.1 \| NA \| 191.748 \| 122.529 \| 16.0 \| \| **Exponential**  **(RANDOM treatment effects)** \| 124.6 \| 3.848 \| 97.973 \| 28.755 \| 27.2 \| \| **Restricted cubic spline**  **(common treatment effects; 3 knots)** \| 203.9 \| NA \| 187.663 \| 118.445 \| 16.9 \| \| **Restricted cubic spline**  **(random treatment effects; 3 knots)** \| 124.3 \| 3.997 \| 98.010 \| 28.792 \| 27.0 \| \| **Non-parametric monotonically up**  **(common treatment effects)** \| 215.9 \| NA \| 200.622 \| 131.403 \| 15.8 \| \| **Non-parametric monotonically up**  **(RANDOM treatment effects)** \| 125.2 \| 5.958 \| 97.848 \| 28.630 \| 28.0 \|   **Table 3.5.** Models fit comparison (Frequency)   \| **Model** \| **DIC** \| **SD** \| **Deviance** \| **Residual deviance** \| **pD** \| \| --- \| --- \| --- \| --- \| --- \| --- \| \| **Emax**  **(common treatment effects)** \| 209.0 \| NA \| 193.624 \| 124.406 \| 16.0 \| \| **Emax**  **(RANDOM treatment effects)** \| 128.1 \| 1.930 \| 99.369 \| 30.150 \| 29.2 \| \| **Linear**  **(common treatment effects)** \| 269.9 \| NA \| 254.555 \| 185.337 \| 16.1 \| \| **Linear**  **(random treatment effects)** \| 124.3 \| 4.807 \| 97.841 \| 28.622 \| 27.3 \| \| **Exponential**  **(common treatment effects)** \| 206.8 \| NA \| 191.783 \| 122.565 \| 15.8 \| \| **Exponential**  **(RANDOM treatment effects)** \| 123.8 \| 3.850 \| 97.901 \| 28.683 \| 26.6 \| \| **Restricted cubic spline**  **(common treatment effects; 3 knots)** \| 186.8 \| NA \| 170.306 \| 101.088 \| 17.2 \| \| **Restricted cubic spline**  **(random treatment effects; 3 knots)** \| 124.1 \| 3.771 \| 98.086 \| 28.868 \| 26.8 \| \| **Non-parametric monotonically up**  **(common treatment effects)** \| 206.4 \| NA \| 190.577 \| 121.358 \| 16.5 \| \| **Non-parametric monotonically up**  **(RANDOM treatment effects)** \| 124.7 \| 4.343 \| 97.907 \| 28.688 \| 27.4 \|   **Table 3.6.** Models fit comparison (Exercises)   \| **Model** \| **DIC** \| **SD** \| **Deviance** \| **Residual deviance** \| **pD** \| \| --- \| --- \| --- \| --- \| --- \| --- \| \| **Emax**  **(common treatment effects)** \| 179.6 \| NA \| 163.846 \| 94.628 \| 16.3 \| \| **Emax**  **(RANDOM treatment effects)** \| 123.4 \| 3.441 \| 97.956 \| 28.738 \| 26.2 \| \| **Linear**  **(common treatment effects)** \| 190.5 \| NA \| 175.231 \| 106.012 \| 16.0 \| \| **Linear**  **(random treatment effects)** \| 123.9 \| 3.601 \| 98.020 \| 28.801 \| 26.6 \| \| **Exponential**  **(common treatment effects)** \| 175.4 \| NA \| 159.512 \| 90.294 \| 16.6 \| \| **Exponential**  **(RANDOM treatment effects)** \| 124.5 \| 3.851 \| 98.077 \| 28.859 \| 27.0 \| \| **Restricted cubic spline**  **(common treatment effects; 3 knots)** \| 169.0 \| NA \| 152.631 \| 83.412 \| 17.1 \| \| **Restricted cubic spline**  **(random treatment effects; 3 knots)** \| 124.2 \| 3.408 \| 98.412 \| 29.193 \| 26.5 \| \| **Non-parametric monotonically up**  **(common treatment effects)** \| 166.7 \| NA \| 149.045 \| 79.826 \| 18.2 \| \| **Non-parametric monotonically up**  **(RANDOM treatment effects)** \| 125.1 \| 4.050 \| 98.675 \| 29.457 \| 27.0 \|   **Table 3.7.** Models fit comparison (Intensity)   \| **Model** \| **DIC** \| **SD** \| **Deviance** \| **Residual deviance** \| **pD** \| \| --- \| --- \| --- \| --- \| --- \| --- \| \| **Emax**  **(common treatment effects)** \| 207.9 \| NA \| 191.513 \| 122.295 \| 17.0 \| \| **Emax**  **(RANDOM treatment effects)** \| 124.8 \| 4.094 \| 98.196 \| 28.978 \| 27.2 \| \| **Linear**  **(common treatment effects)** \| 213.8 \| NA \| 198.584 \| 129.366 \| 15.9 \| \| **Linear**  **(random treatment effects)** \| 125.2 \| 4.293 \| 98.158 \| 28.940 \| 27.5 \| \| **Exponential**  **(common treatment effects)** \| 206.9 \| NA \| 122.382 \| 191.600 \| 16.0 \| \| **Exponential**  **(RANDOM treatment effects)** \| 124.2 \| 3.855 \| 98.142 \| 28.924 \| 26.8 \| \| **Restricted cubic spline**  **(common treatment effects; 3 knots)** \| 211.3 \| NA \| 194.784 \| 125.566 \| 17.1 \| \| **Restricted cubic spline**  **(random treatment effects; 3 knots)** \| 124.8 \| 4.212 \| 98.147 \| 28.929 \| 27.2 \| \| **Non-parametric monotonically up**  **(common treatment effects)** \| 182.8 \| NA \| 166.714 \| 97.496 \| 16.8 \| \| **Non-parametric monotonically up**  **(RANDOM treatment effects)** \| 125.0 \| 4.707 \| 98.598 \| 29.379 \| 27.3 \| |
| --- | --- | --- | --- | --- | --- | --- | --- | --- | --- | --- | --- | --- | --- | --- | --- | --- | --- | --- | --- | --- | --- | --- | --- | --- | --- | --- | --- | --- | --- | --- | --- | --- | --- | --- | --- | --- | --- | --- | --- | --- | --- | --- | --- | --- | --- | --- | --- | --- | --- | --- | --- | --- | --- | --- | --- | --- | --- | --- | --- | --- | --- | --- | --- | --- | --- | --- | --- | --- | --- | --- | --- | --- | --- | --- | --- | --- | --- | --- | --- | --- | --- | --- | --- | --- | --- | --- | --- | --- | --- | --- | --- | --- | --- | --- | --- | --- | --- | --- | --- | --- | --- | --- | --- | --- | --- | --- | --- | --- | --- | --- | --- | --- | --- | --- | --- | --- | --- | --- | --- | --- | --- | --- | --- | --- | --- | --- | --- | --- | --- | --- | --- | --- | --- | --- | --- | --- | --- | --- | --- | --- | --- | --- | --- | --- | --- | --- | --- | --- | --- | --- | --- | --- | --- | --- | --- | --- | --- | --- | --- | --- | --- | --- | --- | --- | --- | --- | --- | --- | --- | --- | --- | --- | --- | --- | --- | --- | --- | --- | --- | --- | --- | --- | --- | --- | --- | --- | --- | --- | --- | --- | --- | --- | --- | --- | --- | --- | --- | --- | --- | --- | --- | --- | --- | --- | --- | --- | --- | --- | --- | --- | --- | --- | --- | --- | --- | --- | --- | --- | --- | --- | --- | --- | --- | --- | --- | --- | --- | --- | --- | --- | --- | --- | --- | --- | --- | --- | --- | --- | --- | --- | --- | --- | --- | --- | --- | --- | --- | --- | --- | --- | --- | --- | --- | --- | --- | --- | --- | --- | --- | --- | --- | --- | --- | --- | --- | --- | --- | --- | --- | --- | --- | --- | --- | --- | --- | --- | --- | --- | --- | --- | --- | --- | --- | --- | --- | --- | --- | --- | --- | --- | --- | --- | --- | --- | --- | --- | --- | --- | --- | --- | --- | --- | --- | --- | --- | --- | --- | --- | --- | --- | --- | --- | --- | --- | --- | --- | --- | --- | --- | --- | --- | --- | --- | --- | --- | --- | --- | --- | --- | --- | --- | --- | --- | --- | --- | --- | --- | --- | --- | --- | --- | --- | --- | --- | --- | --- | --- | --- | --- | --- | --- | --- | --- | --- | --- | --- | --- | --- | --- | --- | --- | --- | --- | --- | --- | --- | --- | --- | --- | --- | --- | --- | --- | --- | --- | --- | --- | --- | --- | --- | --- | --- | --- | --- | --- | --- | --- | --- | --- | --- | --- | --- | --- | --- | --- | --- | --- | --- | --- | --- | --- | --- | --- | --- | --- | --- | --- | --- | --- | --- | --- | --- | --- | --- | --- | --- | --- | --- | --- | --- | --- | --- | --- | --- | --- | --- | --- | --- | --- | --- | --- | --- | --- | --- | --- | --- | --- | --- | --- | --- | --- | --- | --- | --- | --- | --- | --- | --- | --- | --- | --- | --- | --- | --- | --- | --- | --- | --- | --- | --- | --- | --- |

*Note.* DIC = Deviance Information Criterion; SD = Between-study Standard Deviation; pD: Number of estimated parameters; NA = Not Applicable. The SD is presented as the main value and (95% Credible Intervals).

Further to model fit indices, deviance plots showing the contribution of each data point to the residual deviance are also useful to confirm the robustness of model selection (Pedder H, 2021). Each data point should contribute about 1 to the posterior mean deviance, which indicates good model fit (Dias S, et al., 2013). The deviance plot for treatment effects (Supplementary File X) confirm the robustness of our model selection.

### **Supplementary Figure 5**. The deviance plot for treatment effects.


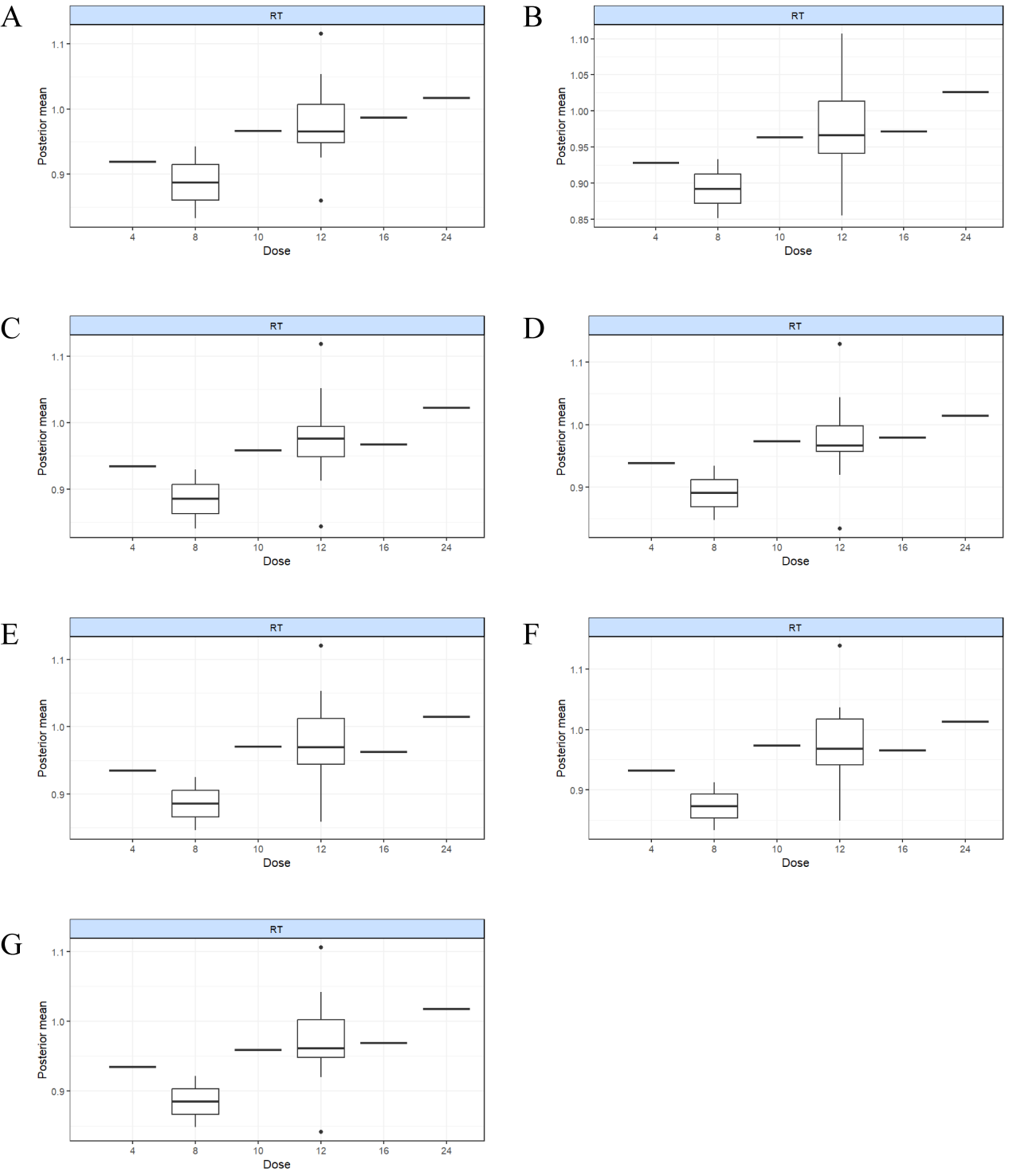


**Supplementary Figure 5**. The deviance plot for treatment effects. (A): Sets; (B): Period; (C): Reps/week; (D): Reps; (E): Times; (F): Exercises; (G): Intensity

# Supplementary file 5: Characteristics of included studies

### **Supplementary Table 4.** Methods of data coding and management

Table.4.1 Methods of recording exercise dose elements

| values | Recording methods | eg. (Frequency). | Record |
| --- | --- | --- | --- |
| fixed dose values | original values | 3 times/week | 3 |
| certain range | average value of the range | 2-3 times/week | 2.5 |

Table.4.2 The Borg Rating of Perceived Exertion (RPE) Scale

| Grade 15 | | Grade 10 | |
| --- | --- | --- | --- |
| 6 |  | 0 | Nothing at all |
| 7 | Very, very light | 0.5 | Very, very weak |
| 8 |  | 1 | Very weak |
| 9 | Very light | 2 | weak |
| 10 |  | 3 | moderate |
| 11 | Fairly light | 4 | Somewhat strong |
| 12 |  | 5 | strong |
| 13 | Somewhat hard | 6 |  |
| 14 |  | 7 | Very strong |
| 15 | hard | 8 |  |
| 16 |  | 9 |  |
| 17 | Very hard | 10 | Very, very strong |
| 18 |  | ≥10 | Maximal |
| 19 | Very, very hard |  |  |
| 20 |  |  |  |

Table.4.3 1RM-Repetition Table

| Repetitions | %1RM |
| --- | --- |
| 1 | 100 |
| 2 | 95 |
| 3 | 93 |
| 4 | 90 |
| 5 | 87 |
| 6 | 85 |
| 7 | 83 |
| 8 | 80 |
| 9 | 77 |
| 10 | 75 |
| 11 | 70 |
| 12 | 67 |
| 15 | 65 |

Table.4.4 Methods of Estimating Intensity of Cardiorespiratory and Resistance Exercise

| Intensity | Perceived Exertion (Rating on 6-20 RPE Scale) | % ONE Repetition Maximum |
| --- | --- | --- |
| Very light | Very light (RPE≤9) | ＜30 |
| Light | Very light to fairly light (RPE 9-11) | 30—＜50 |
| Moderate | fairly light to somewhat hard (RPE 12-13) | 50—＜70 |
| Vigorous | somewhat hard to very hard (RPE 12-13) | 70—＜85 |
| Near maximal to maximal | ≥Very hard (RPE ≥18) | ≥85 |

# Supplementary file 6: Characteristics of included studies

This supplementary file provides the datasets (i.e., at global exercise and agent levels) used in this study. The *studyID* indicates the author’s name 、the year of publication and study number. The *agent* indicates groups in studies. The *y* indicates the average of all values of research variable. The *n* indicates the number of participants in the study.The *se* indicates the standard error of the mean. The *sd_session* indicates the standard deviation of the mean. The *frequency* is the number of days per week that participants were participated in physical activity.The *Period* indicates exercise period in the study. The *Set* indicates the group of doses by approximation. The *Reps* indicates the times of repetition by approximation. The *Intensity* indicates the percentage of participants completed OneRepMax. The *Reps/week* indicates the number of weekly repetitions by approximation. The *Exercises* indicates the number of exercises per set that participants were involved in physical activity.

| **studyID** | **agent** | **y** | **n** | **se** | **sd** | **Frequency** | **Period** | **Set** | **Reps** | **Intensity** | **Reps/week** | **Exercises** |
| --- | --- | --- | --- | --- | --- | --- | --- | --- | --- | --- | --- | --- |
| Silva ACD, et al(2024)^1^ | RT | 31.68 | 14 | 0.638754368 | 2.39 | 3 | 12 | 3 | 10 | 75 | 990 | 11 |
|  | CON | 18.43 | 11 | 0.820110857 | 2.72 | 0 | 0 | 0 | 0 | 0 | 0 | 0 |
| Silva ACD, et al(2024)^1^ | RT | 29.95 | 20 | 0.44274146 | 1.98 | 3 | 12 | 3 | 10 | 75 | 990 | 11 |
|  | CON | 17.94 | 17 | 0.6596969 | 2.72 | 0 | 0 | 0 | 0 | 0 | 0 | 0 |
| Silva ACD, et al(2024)^1^ | RT | 22.38 | 5 | 1.721772343 | 3.85 | 3 | 12 | 3 | 10 | 75 | 990 | 11 |
|  | CON | 18.67 | 4 | 3.045 | 6.09 | 0 | 0 | 0 | 0 | 0 | 0 | 0 |
| Xiao Y, et al(2023)^2^ | RT | 21.50 | 30 | 1.040672859 | 5.70 | 6 | 4 | 3 | 30 | 75 | 2160 | 4 |
|  | CON | 19.40 | 30 | 1.022415441 | 5.60 | 0 | 0 | 0 | 0 | 0 | 0 | 0 |
| Chen HT, et al(2018)^3^ | RT | 21.50 | 17 | 1.042903188 | 4.30 | 2 | 8 | 3 | 10 | 65 | 660 | 11 |
|  | CON | 17.30 | 16 | 1.065 | 4.26 | 0 | 0 | 0 | 0 | 0 | 0 | 0 |
| Lee YH, et al(2021)^4^ | RT | 20.35 | 15 | 3.529578823 | 13.67 | 3 | 12 | 3 | 10 | 60 | 720 | 8 |
|  | CON | 18.11 | 12 | 5.909180005 | 20.47 | 0 | 0 | 0 | 0 | 0 | 0 | 0 |
| Vikberg S, et al(2019)^5^ | RT | 32.00 | 31 | 1.921776732 | 10.70 | 3 | 10 | 3 | 11 | 75 | 792 | 8 |
|  | CON | 30.50 | 34 | 1.817885003 | 10.60 | 0 | 0 | 0 | 0 | 0 | 0 | 0 |
| Dong ZJ, et al(2019)^6^ | RT | 26.03 | 21 | 0.840138877 | 3.85 | 3 | 12 | 10 | 10 | 50 | 900 | 3 |
|  | CON | 21.34 | 20 | 1.377417874 | 6.16 | 0 | 0 | 0 | 0 | 0 | 0 | 0 |
| Yamada M, et al(2019)^7^ | RT | 24.67 | 8 | 0.922774349 | 2.61 | 2 | 12 | 3 | 20 | 30 | 840 | 7 |
|  | CON | 16.28 | 8 | 1.025304833 | 2.90 | 0 | 0 | 0 | 0 | 0 | 0 | 0 |
| Gadelha AB, et al(2021)^8^ | RT | 27.44 | 37 | 0.937074228 | 5.70 | 3 | 24 | 3 | 10 | 30 | 1080 | 12 |
|  | CON | 20.28 | 28 | 0.888216512 | 4.70 | 0 | 0 | 0 | 0 | 0 | 0 | 0 |
| Seo MW, et al(2021)^9^ | RT | 24.30 | 12 | 0.649519053 | 2.25 | 3 | 16 | 4 | 12 | 70 | 2448 | 17 |
|  | CON | 17.30 | 10 | 1.141582235 | 3.61 | 0 | 0 | 0 | 0 | 0 | 0 | 0 |
| Chien YH, et al(2022)^10^ | RT | 19.10 | 20 | 1.095673309 | 4.90 | 3 | 12 | 3 | 12 | 70 | 540 | 5 |
|  | CON | 16.50 | 20 | 1.207476708 | 5.40 | 0 | 0 | 0 | 0 | 0 | 0 | 0 |
| de Sá Souza H, et al(2022)^11^ | RT | 30.46 | 14 | 2.375952441 | 8.89 | 3 | 12 | 2 | 11 | 65 | 528 | 8 |
|  | CON | 20.62 | 14 | 1.167931627 | 4.37 | 0 | 0 | 0 | 0 | 0 | 0 | 0 |
| Chen HT, et al(2017)^12^ | RT | 23.50 | 15 | 1.884851895 | 7.30 | 2 | 8 | 3 | 10 | 65 | 600 | 10 |
|  | CON | 19.70 | 15 | 2.349609897 | 9.10 | 0 | 0 | 0 | 0 | 0 | 0 | 0 |
| Liao CD, et al(2017)^13^ | RT | 24.49 | 25 | 1.148 | 5.74 | 3 | 12 | 3 | 16 | 70 | 864 | 6 |
|  | CON | 20.64 | 21 | 1.31148952 | 6.01 | 0 | 0 | 0 | 0 | 0 | 0 | 0 |

# List of included studies

1. Silva ACD, Mapa V, Ferreira-Júnior JB, Oliveira EC, Becker LK, Rosse I, Coelho DB. Progressive strength training can reverse sarcopenia stage in middle-aged and older adults regardless of their genetic profile. Arch Gerontol Geriatr. 2024;117:105182. doi: 10.1016/j.archger.2023.105182.
2. Xiao Y, Song D, Fu N, Zhang L, Zhang Y, Shen R, Wang S, Maitiabula G, Zhou D, Liu S, Wang H, Gao X, Wang X. Effects of resistance training on sarcopenia in patients with intestinal failure: A randomized controlled trial. Clin Nutr. 2023;42(10):1901-1909. doi: 10.1016/j.clnu.2023.07.013.
3. Chen HT, Wu HJ, Chen YJ, Ho SY, Chung YC. Effects of 8-week kettlebell training on body composition, muscle strength, pulmonary function, and chronic low-grade inflammation in elderly women with sarcopenia. Exp Gerontol. 2018;112:112-118. doi: 10.1016/j.exger.2018.09.015.
4. Lee YH, Lee PH, Lin LF, Liao CD, Liou TH, Huang SW. Effects of progressive elastic band resistance exercise for aged osteosarcopenic adiposity women. Exp Gerontol. 2021;147:111272. doi: 10.1016/j.exger.2021.111272.
5. Vikberg S, Sörlén N, Brandén L, Johansson J, Nordström A, Hult A, Nordström P. Effects of Resistance Training on Functional Strength and Muscle Mass in 70-Year-Old Individuals With Pre-sarcopenia: A Randomized Controlled Trial. J Am Med Dir Assoc. 2019;20(1):28-34. doi: 10.1016/j.jamda.2018.09.011.
6. Dong ZJ, Zhang HL, Yin LX. Effects of intradialytic resistance exercise on systemic inflammation in maintenance hemodialysis patients with sarcopenia: a randomized controlled trial. Int Urol Nephrol. 2019;51(8):1415-1424. doi: 10.1007/s11255-019-02200-7.
7. Yamada M, Kimura Y, Ishiyama D, Nishio N, Otobe Y, Tanaka T, Ohji S, Koyama S, Sato A, Suzuki M, Ogawa H, Ichikawa T, Ito D, Arai H. Synergistic effect of bodyweight resistance exercise and protein supplementation on skeletal muscle in sarcopenic or dynapenic older adults. Geriatr Gerontol Int. 2019;19(5):429-437. doi: 10.1111/ggi.13643.
8. Gadelha AB, Cesari M, Corrêa HL, Neves RVP, Sousa CV, Deus LA, Souza MK, Reis AL, Moraes MR, Prestes J, Simões HG, Andrade RV, Melo GF, Rosa TS. Effects of pre-dialysis resistance training on sarcopenia, inflammatory profile, and anemia biomarkers in older community-dwelling patients with chronic kidney disease: a randomized controlled trial. Int Urol Nephrol. 2021 Oct;53(10):2137-2147. doi: 10.1007/s11255-021-02799-6.
9. Seo MW, Jung SW, Kim SW, Lee JM, Jung HC, Song JK. Effects of 16 Weeks of Resistance Training on Muscle Quality and Muscle Growth Factors in Older Adult Women with Sarcopenia: A Randomized Controlled Trial. Int J Environ Res Public Health. 2021;18(13):6762. doi: 10.3390/ijerph18136762.
10. Chien YH, Tsai CJ, Wang DC, Chuang PH, Lin HT. Effects of 12-Week Progressive Sandbag Exercise Training on Glycemic Control and Muscle Strength in Patients with Type 2 Diabetes Mellitus Combined with Possible Sarcopenia. Int J Environ Res Public Health. 2022 Nov 15;19(22):15009. doi: 10.3390/ijerph192215009. PMID: 36429728; PMCID: PMC9690442.
11. de Sá Souza H, de Melo CM, Piovezan RD, Miranda REEPC, Carneiro-Junior MA, Silva BM, Thomatieli-Santos RV, Tufik S, Poyares D, D'Almeida V. Resistance Training Improves Sleep and Anti-Inflammatory Parameters in Sarcopenic Older Adults: A Randomized Controlled Trial. Int J Environ Res Public Health. 2022;19(23):16322. doi: 10.3390/ijerph192316322.
12. Chen HT, Chung YC, Chen YJ, Ho SY, Wu HJ. Effects of Different Types of Exercise on Body Composition, Muscle Strength, and IGF-1 in the Elderly with Sarcopenic Obesity. J Am Geriatr Soc. 2017;65(4):827-832. doi: 10.1111/jgs.14722.
13. Liao CD, Tsauo JY, Lin LF, Huang SW, Ku JW, Chou LC, Liou TH. Effects of elastic resistance exercise on body composition and physical capacity in older women with sarcopenic obesity: A CONSORT-compliant prospective randomized controlled trial. Medicine (Baltimore). 2017;96(23):e7115. doi: 10.1097/MD.0000000000007115.

# References

1 [ter Veer E, van Oijen MGH, van Laarhoven HWM. The Use of (Network) Meta-Analysis in Clinical Oncology.](http://paperpile.com/b/b8T1z2/YP82p) *[Front Oncol](http://paperpile.com/b/b8T1z2/YP82p)* [2019;](http://paperpile.com/b/b8T1z2/YP82p)**[9](http://paperpile.com/b/b8T1z2/YP82p)**[:822. doi:](http://paperpile.com/b/b8T1z2/YP82p)[10.3389/fonc.2019.00822](http://dx.doi.org/10.3389/fonc.2019.00822)

2 [Wheeler DC, Hickson DA, Waller LA. Assessing local model adequacy in Bayesian hierarchical models using the partitioned deviance information criterion. Computational Statistics & Data Analysis. 2010;](http://paperpile.com/b/b8T1z2/jIaJT)**[54](http://paperpile.com/b/b8T1z2/jIaJT)**[:1657–71. doi:](http://paperpile.com/b/b8T1z2/jIaJT)[10.1016/j.csda.2010.01.025](http://dx.doi.org/10.1016/j.csda.2010.01.025)

3 Pedder H. MBNMAdose: An R package for incorporating dose-response information into Network Meta-Analysis. In: Evidence Synthesis and Meta-Analysis in R Conference 2021. 2021. <https://research-information.bris.ac.uk/en/publications/mbnmadose-an-r-package-for-incorporating-dose-response-information>

4 [Dias S, Sutton AJ, Ades AE,](http://paperpile.com/b/b8T1z2/6GsS) *[et al.](http://paperpile.com/b/b8T1z2/6GsS)* [Evidence synthesis for decision making 2: a generalized linear modeling framework for pairwise and network meta-analysis of randomized controlled trials.](http://paperpile.com/b/b8T1z2/6GsS) *[Med Decis Making](http://paperpile.com/b/b8T1z2/6GsS)* [2013;](http://paperpile.com/b/b8T1z2/6GsS)**[33](http://paperpile.com/b/b8T1z2/6GsS)**[:607–17. doi:](http://paperpile.com/b/b8T1z2/6GsS)[10.1177/0272989X12458724](http://dx.doi.org/10.1177/0272989X12458724).

[1] PEDDER H, DIAS S, BENNETTS M, et al. Modelling time-course relationships with multiple treatments: Model-based network meta-analysis for continuous summary outcomes [J]. Res Synth Methods, 2019, 10(2): 267-86.

[2] EVANS N J. Assessing the practical differences between model selection methods in inferences about choice response time tasks [J]. Psychon B Rev, 2019, 26(4): 1070-98.

[3] HAMZA T, CIPRIANI A, FURUKAWA T A, et al. A Bayesian dose-response meta-analysis model: A simulations study and application [J]. Stat Methods Med Res, 2021, 30(5): 1358-72.

[4] NUNEZ E, STEYERBERG E W, NUNEZ J. [Regression modeling strategies] [J]. Rev Esp Cardiol, 2011, 64(6): 501-7.
